# Experimental Studies on State Self-Objectification: A Review and an Integrative Process Model

**DOI:** 10.3389/fpsyg.2018.01268

**Published:** 2018-08-13

**Authors:** Rotem Kahalon, Nurit Shnabel, Julia C. Becker

**Affiliations:** ^1^Tel-Aviv University, The School of Psychological Science, Tel-Aviv, Israel; ^2^School of Human Science, Institute of Psychology, Osnabrück University, Osnabrück, Germany

**Keywords:** objectification theory, self-objectification, stereotype threat, schema activation, appearance monitoring, appearance standards, an integrative process model, experimental research

## Abstract

This paper provides an organizing framework for the experimental research on the effects of state self-objectification on women. We explain why this body of work, which had grown rapidly in the last 20 years, departs from the original formulation of objectification theory (Fredrickson and Roberts, 1997). We compare the different operationalizations of state self-objectification and examine how they map onto its theoretical definition, concluding that the operationalizations have focused mostly on one component of this construct (concerns about one's physical appearance) while neglecting others (adopting a third-person perspective and treating oneself as a dehumanized object). We review the main findings of studies that experimentally induced state self-objectification and examined its affective, motivational, behavioral, cognitive, and physiological outcomes. We note that three core outcomes of this state as specified by objectification theory (safety anxiety, reduced flow experiences, and awareness of internal body states) have hardly been examined so far. Most importantly, we introduce an integrative process model, suggesting that the reported effects are triggered by four different mechanisms: appearance monitoring, experience of discrepancy from appearance standards, stereotype threat, and activation of the “sex object” schema. We propose strategies for distinguishing between these mechanisms and explain the theoretical and practical importance of doing so.

Several feminist theorists have argued that Western culture promotes sexual objectification of the female body—namely, treating women as bodies that exist, and are primarily valued, for the use and pleasure of others (e.g., De Beauvoir, 1952; Berger, 1972; Dworkin, 1974; Orbach, 1978; Baker Miller, 1986; Ussher, 1989; Wolf, 1991; Bordo, 1993; Nussbaum, 1999). The sexual objectification of a woman involves separating her body, body parts, or sexual functions from her identity (thus reducing her to the status of a mere instrument), or regarding them as though they can represent her as a whole (Bartky, 1990). Women's sexual objectification in Western society is enacted in many ways, ranging from sexual violence (Brownmiller, 1975) to sexualized evaluation (Kaschak, 1992). These occur both in actual interpersonal and social interactions (e.g., street harassment) and in the visual media (e.g., ads depicting body parts of female models) (Fredrickson and Roberts, 1997).

Building on the insights of feminist theorizing, objectification theory (Fredrickson and Roberts, 1997) offers a social psychological framework for understanding the consequences for women's well-being of living in a culture that objectifies the female body. According to this theory, girls and women in Western society are socialized to view themselves as objects meant to be looked at and evaluated based on their appearance. This process of *self-objectification—*adopting an observer's perspective on the physical self—leads women to a form of self-consciousness characterized by monitoring their body's appearance. Self-objectification can be conceptualized as a *trait*, representing the extent to which women have internalized a third-person perspective toward their body and are chronically preoccupied with their appearance (i.e., across situations). It can also be conceptualized as a *state* (i.e., a temporary condition), representing women's situational awareness of an actual or imaginary observer's perspective toward their bodies and a subsequent preoccupation with their appearance (Fredrickson et al., 1998). This form of self-consciousness increases women's experience of shame (due to their failure to meet internalized ideal beauty standards) as well as anxiety regarding their appearance and safety (i.e., fear of sexual assault). It also reduces their experience of “flow” (peak motivational states in which one is fully absorbed in a rewarding activity; Csikszentmihalyi, 1990) and awareness of internal bodily states (access to inner physical experiences; e.g., hunger cues or sexual arousal). The accumulation of these negative experiences contributes to the disproportionately high rate among women of three psychological disorders: unipolar depression, sexual dissatisfaction and dysfunctions, and eating disorders. These mental health risks intensify in early adolescence and lessen in late middle age, when women step in and out of “the objectification limelight” (Fredrickson and Roberts, 1997).

Because objectification theory takes as its starting point that women are chronically sexually objectified in Westernized societies (Calogero, 2011), and due to the theory's broad scope in terms of: (a) its focus on mental health outcomes and changes across the life course, and (b) its emphasis on the accumulating effects of objectification, much of the research conducted within its framework has focused on studying the antecedents and consequences of trait self-objectification (e.g., its association with disordered eating, such as caloric restriction and bulimic behaviors; Moradi et al., 2005; Calogero, 2009). Also, many of the theory's arguments can ideally be tested using correlational or longitudinal designs (e.g., examining how self-objectification changes across the life span; Tiggemann and Lynch, 2001) rather than through cross-sectional, de-contextualized lab experiments (Tiggemann, 2011).

However, understanding the immediate effects of state self-objectification, as well as the specific social-psychological mechanisms driving them, can be best achieved through controlled lab experiments, which allow causal inference (Falk and Heckman, 2009). The present paper reviews and offers a novel organizing framework for the existing body of experimental work on state self-objectification, which is summarized in Table A1 in Appendix. The purpose of this organizing framework is not to criticize or introduce changes into objectification theory, which has a very broad scope and which focuses primarily on trait self-objectification. Rather, the proposed framework aims to reorganize and reinterpret the findings of existing experimental studies, in order to provide a rigorous answer to a specific question: *What happens to women (and men) in objectifying situations that make them focus on their appearance?*

The first and foremost of these studies, the seminal paper “That Swimsuit Becomes You” (Fredrickson et al., 1998), attempted to experimentally test hypotheses derived from objectification theory through examining the effects of trying on a swimsuit (as compared to a sweater) on female and male participants. One key finding was that the swimsuit condition (intended to situationally increase women's view of themselves as sexual objects) led to impaired math performance among female but not among male participants, presumably because it directed their attentional resources to their bodies, which limited their available cognitive resources (Fredrickson et al., 1998). This finding was interpreted as “consistent with the claim of objectification theory that these consequences appear to be unique to young women socialized in a culture that sexually objectifies the female body” (Fredrickson et al., 1998; p. 280), and prompted a vast body of experimental research.

Although the body of experimental research has clearly been *inspired* by objectification theory, we argue that it should not be viewed as a test of the full-blown theory, since experiments are not the ideal methodology for doing so. As stated above, experiments are ideal for the purpose of rigorously examining the immediate effects of state self-objectification—not the lifelong consequences of the chronic induction of this state among women in Western society, which results in heightened trait self-objectification (Moradi et al., 2005). The paper “The Swimsuit Becomes Us All” (Hebl et al., 2004) illustrates our argument. Hebl et al. used the swimsuit-sweater paradigm with one modification—male participants tried on revealing Speedos instead of swim trunks—and found that both men and women had worse math performance in the swimsuit condition. This finding refutes Fredrickson et al.'s (1998) conclusion that objectifying situations lead to performance decrements *solely* among women. However, it does not refute objectification theory (Fredrickson and Roberts, 1997) itself, because it is possible that whereas both men and women show negative responses to objectifying situations induced in the lab, the real-life exposure to such situations is much less frequent for men (e.g., most American men have never worn revealing Speedos) than for women, for whom they compound to larger negative outcomes over time.

We therefore claim that the question “*What happens to women and men in objectifying situations?*” can be discussed independently of the question “*Is objectification theory true?*” Indeed, experimental studies that examined the effects of objectifying situations in the lab constitute a growing body of work that has *departed from* objectification theory. To illustrate, Calogero (2013) demonstrated that objectifying situations decrease women's support of taking action to reduce gender inequality, an outcome variable that objectification theory is silent on. Thus, this body of experimental work should be analyzed based on its own merit, in relationship to relevant social psychological theories besides objectification theory (as the prism of one single theory is too narrow). The present paper offers such an analysis: Besides reviewing the experimental literature on self-objectification, we critically identify gaps in the literature and develop an integrative theoretical process model, pointing to specific causal mechanisms. Our model puts forward an organizing framework for the existing body of work, which could be tested in future work.

In the next sections, we first fully define state self-objectification, review the existing operationalizations of this state, and examine how they map onto its theoretical definition. We note that with only few exceptions, the lab studies that examined state self-objectification focused on one key component of this theoretical construct (as defined by objectification theory), while neglecting two additional key components. Next, we review the main findings of the studies that experimentally induced state self-objectification. Here we note that three of the key outcomes of this state as specified in objectification theory were ignored in these studies.

Most importantly, we develop a model explaining how the outcomes of state self-objectification observed in the literature may be accounted for by the four psychological mechanisms schematically presented in Figure 1. As we explain in detail later, two of these mechanisms—appearance monitoring (Fredrickson et al., 1998) and experience of discrepancy from appearance standards (Quinn et al., 2011)—have been discussed in the literature yet should be further distinguished from each other theoretically and empirically. The third mechanism, stereotype threat (concern about confirming the negative stereotype about one's ingroup; Steele, 1997), has been considered to be ruled out based on evidence, but that evidence is insufficient, as we explain later. The fourth mechanism, activation (priming) of the “sex object” schema, has not been considered in the literature and is discussed for the first time here. We demonstrate that some of the outcomes of state self-objectification reported in the literature (e.g., increased support for existing gender arrangements; Calogero, 2013) are best accounted for by this mechanism and cannot be accounted for by the other mechanisms. After presenting our model, we propose a series of strategies for empirically distinguishing between the four mechanisms. We conclude by discussing the theoretical and practical implications of the proposed model.

**Figure 1:**
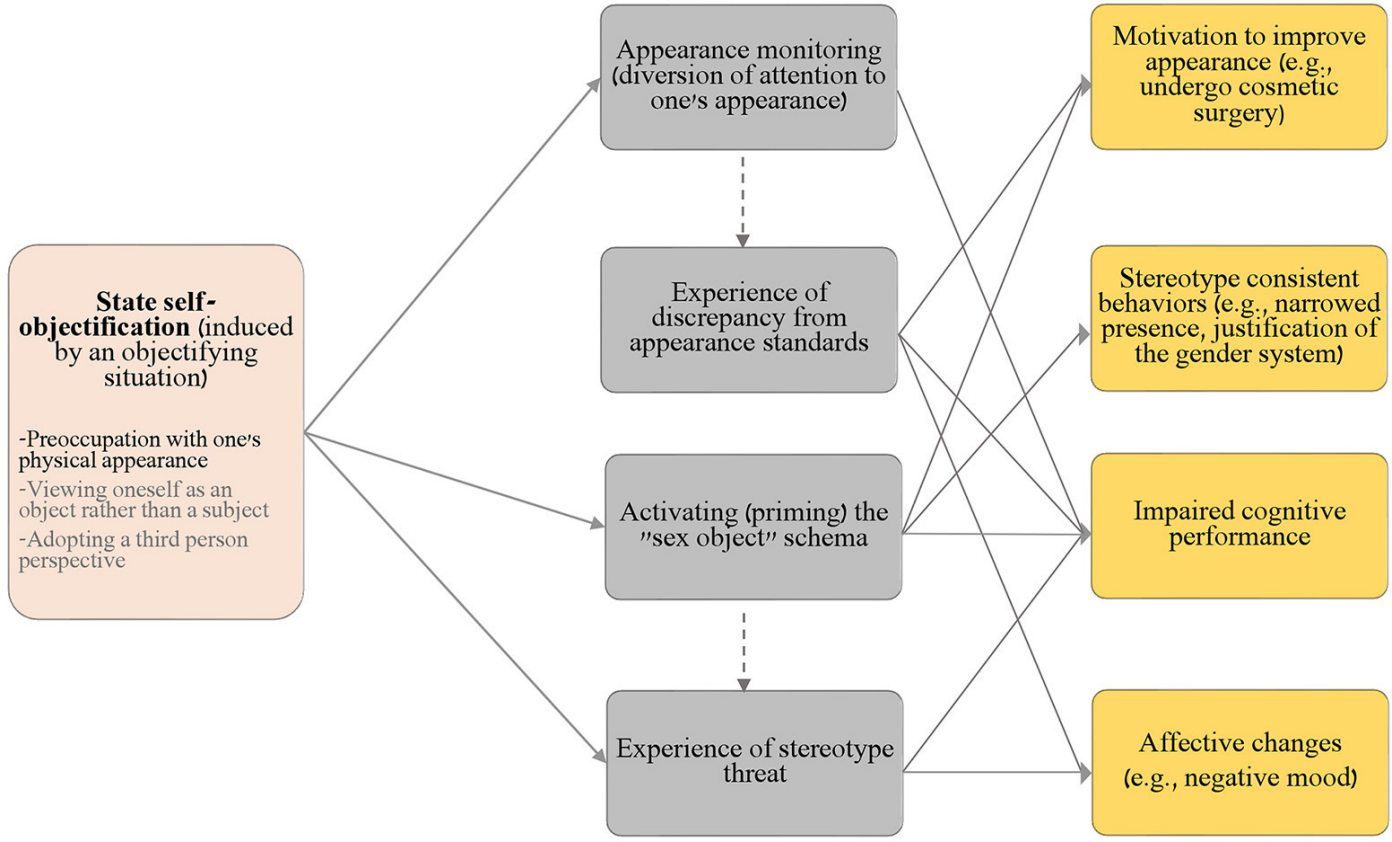
The proposed integrative process model of state self-objectification in women. According to the model, the induction of state self-objectification through objectifying situations (e.g., trying on a swimsuit) can trigger four social-psychological processes (mechanisms), which lead to the host of affective responses, and changes in cognitive performance, motivations, and behaviors that are reported in the experimental literature on this state. In the state self-objectification box, aspects of self-objectification for which additional empirical evidence is required appear in a light gray font; the empirically established aspect of this state appears in a black font.

## State self-objectification: theoretical and operational definitions

### Definition

According to objectification theory, self-objectification is the psychological construct linking women's involvement in cultural practices of sexual objectification (e.g., exposure to catcalls or beauty magazines) and their subsequent experience of four outcomes—heightened shame and anxiety (specifically, appearance anxiety and safety anxiety), and reduced flow and sensitivity to body cues (Fredrickson and Roberts, 1997). Self-objectification has been defined as becoming preoccupied with one's own physical appearance due to the internalization of an objectifying observer's perspective on one's own body (Fredrickson and Roberts, 1997), such that “individuals think about and value their own body more from a third-person perspective, focusing on observable body attributes (e.g., “How do I look?”), rather than from a first-person perspective, focusing on privileged, or non-observable body attributes (e.g., “What am I capable of?” or “How do I feel?”)” (Fredrickson et al., 1998; p. 270).

Other theorists have used somewhat different definitions. Lindner and Tantleff-Dunn (2017) recently proposed an empirically derived definition of self-objectification as: (a) internalizing the observer's perspective on the body, and (b) treating the body as if it is capable of representing the self. However, due to its emphasis on a long-term process of internalization and on global perceptions of the self, this definition is more suitable for the conceptualization of trait rather than state self-objectification (see Chaplin et al., 1988, for the notion that traits are stable, internally caused concepts, which serve to predict interpersonal events from past behavior, whereas states are brief, and caused by external circumstances). Calogero et al. (2011, p. 218), defined state self-objectification as including “the doubling of women's attention […] taking a view of the self from one's own vantage point and from the perspective of another person simultaneously;” “disconnection and distancing between the self and the body” and women's view of themselves “through the lens of the male gaze.” However, given evidence that men, too, experience consequences such as increased body shame and impairment to cognitive performance under objectifying conditions (e.g., Hebl et al., 2004), it was important for us to use a definition that captures the experience of state self-objectification in both genders (e.g., whereas “viewing oneself through the male gaze” cannot describe men's experience of this state). We therefore adopted (Fredrickson et al., 1998) definition, according to which state self-objectification includes three components: preoccupation with one's physical appearance, viewing oneself as an object, and adopting a third-person perspective.

### Measurements

There are three common measures to assess state self-objectification. The most common measure, which is often used as a manipulation check in the lab studies that induce state self-objectification, is the Twenty Statements Test (TST; Noll and Fredrickson, 1998) originally developed to assess people's self-concept (Kuhn and McPartland, 1954). On the TST, respondents are asked to complete 20 statements (or, in shortened versions, 7 or 10 statements) to describe themselves, such that more statements that relate to their body shape or physical attributes are interpreted as indicating greater state self-objectification. The TST was first used for this purpose in Fredrickson et al. (1998) study, and its use has become widespread since then (see Table A1 in Appendix).

An additional measure of state self-objectification that was used in several recent studies (see Table A1 in Appendix) is a modified version of the Self-Objectification Questionnaire (SOQ; Noll and Fredrickson, 1998). Originally developed to measure *trait* self-objectification, the SOQ was adapted by Calogero and Jost (2011) such that respondents are asked to rank 10 body attributes in the order of their importance to their *current* physical self-concept. Five attributes are observable (e.g., weight) and five are non-observable (e.g., health). The sum of the ranks given to the non-observable attributes is subtracted from that of the observable attributes. Hence, higher scores indicate greater importance ascribed to physical appearance.

The Objectified Body Consciousness Scale (OBCS; McKinley and Hyde, 1996) is another common measure of *trait* self-objectification which was modified to measure state self-objectification. Breines et al. (2008) adapted the self-surveillance subscale of the OBCS, which measures habitual body monitoring, to evaluate respondents' situational preoccupation with their appearance (e.g., “right now, I am thinking about how I look”). This measure was used in several experiments, either as a manipulation check or as a dependent variable.

Examining these three measures, we argue that all of them focus on one component of state self-objectification—preoccupation with one's physical appearance. Admittedly, one could argue that preoccupation with one's appearance (the first component of state self-objectification) inextricably involves viewing oneself as an object and adopting a third-person perspective (the two other components of this state). Relevant in this regard is James (1890) distinction between “I” and “me”: “I” represents one's experiential awareness (i.e., self as a subject), whereas “me” represents one's conceptual knowledge about oneself (i.e., self as an object). To illustrate, when a woman thinks about how her body looks (e.g., “I am fat”) rather than how it feels (e.g., “I am hungry”), she is engaging in the type of self-reflection that characterizes the “me” facet of the self. She could therefore be said to be viewing herself as an object and adopting a third-person perspective (as the thought “I am fat” represents her conceptual knowledge of herself which, as opposed to internal “me”' states, is shared by other people). The problem in conceptualizing the three components of state self-objectification as inherently inseparable is that it might create definitional redundancy. Because it is our stance that theoretical definitions should ideally be as concise as possible, minimizing redundancy, our analysis below focuses on the unique, *non-overlapping* parts of the three components.

With regard to viewing oneself as an object (the first missing component), objectified individuals are de-humanized in the sense that they are treated as though they lack the mental states and moral status associated with personhood (Nussbaum, 1999). Studies have revealed that sexually objectified female targets (e.g., partially rather than fully clothed women) were associated with fewer human concepts such as uniquely human emotions (Vaes et al., 2011), attributed less mind and accorded lesser moral status (indicating the denial of personhood; Loughnan et al., 2010), were better recognized by their bodies than by their faces (which, as opposed to bodies, are associated with mental states) (Cikara et al., 2011), and were viewed as less competent, agentic (Heflick and Goldenberg, 2009; Heflick et al., 2011), moral, and warm (Heflick et al., 2011). If so, women in a state of self-objectification should perceive themselves as less agentic, competent, warm, and moral, and as having fewer human attributes or moral entitlement. To date, only one study (Loughnan et al., 2017; Study 1) directly tested these predictions, revealing that under objectifying conditions (i.e., after recalling a situation in which they were sexually objectified) women perceived themselves as lacking in human nature and uniqueness and as less warm and competent, but, unexpectedly, as *more* moral. In addition, one study (Saguy et al., 2010) asked women to rate the extent to which different traits, including ones that represent high agency (e.g., “competent”) and warmth (e.g., “caring”), described their personality; the study found that the manipulation of state self-objectification (knowing that a man will watch their body videotaped from the neck down) had no effect on these ratings. Similarly, Guizzo and Cadinu (2016) found that the exposure to a male (vs. a female) gaze did not affect women's self-perceptions of competence, morality and warmth. Additional research is therefore needed to establish the effects on this aspect (i.e., viewing oneself as an object) of state self-objectification.

The second component of state self-objectification that is not captured by the existing measures is the shift from a first-person to a third-person perspective. Here, it may be useful to consult the literature on visual imagery, in which “perspective” is defined as “a visual viewpoint” (for a review, see Libby and Eibach, 2011). This definition allows disentangling “preoccupation with appearance” from “adopting a third-person perspective.”

To illustrate, imagine an adolescent at the beach who sits down on the sand and notices, when looking down, that she has more tummy rolls in her new position. At this state, she might be preoccupied with her looks yet adopt a first-person (visual) perspective. Building on this understanding, Huebner and Fredrickson (1999) reported that when writing their memories women used more “observer imagery” than men did—namely, they described the event from a third-person rather than a first-person perspective (i.e., visual viewpoint). Yet, the authors acknowledged that such gender differences may stem from gender differences in perspective taking, and therefore “[f]uture empirical work will be needed to determine the extent to which observer imagery is related to self-objectification” (Huebner and Fredrickson, 1999, p. 465). However, in the sole attempt to gain such empirical evidence, the swimsuit-sweater paradigm failed to prompt female participants to report more memories that were “observer oriented” (as seen by an observer/filmed by an external camera) rather than “field oriented” (as seen from the participant's point of view) (Quinn et al., 2011). It also did not lead participants to draw the letter E on their foreheads, so it would appear the right way around to observers (Galinsky et al., 2006) rather than backward, such that it would look correct for the self but backward to observers (Ǝ). Actually, women in the swimsuit condition were the least likely to draw observers' E, suggesting that they were “intensely self-focused during self-objectification” (Quinn et al., 2011, p. 126). The literature on visual imagery (Libby and Eibach, 2011) is highly relevant here, because it makes clear predictions regarding the consequences of adopting a first-person vs. third-person perspective which easily lend themselves to a direct empirical test. Specifically, this literature shows that when people picture situations from a third-person perspective, they focus on the situations' broader abstract meaning in their life, whereas picturing them from a first-person perspective makes people focus on the situations' concrete features. The prediction derived from this literature (that state self-objectification should make women focus on the situation's broader meaning) has not been examined (and is perhaps unlikely to be supported, given that self-objectification is associated with a narrowed focus; Quinn et al., 2011).

Also relevant here is the research on autobiographical memory, according to which past personal events can be remembered from either a field (first-person) or an observer (third-person) perspective (e.g., Nigro and Neisser, 1983). Asking female participants to generate memories about situations in which they were objectified has been used in several studies (e.g., Calogero, 2013) to manipulate state self-objectification; it is possible to examine whether participants have used an observer perspective to a greater extent when generating memories of objectifying as compared to non-objectifying situations. Notably, whereas objectifying situations typically elicit strong affective responses (e.g., of body shame, Fredrickson et al., 1998), research on autobiographical memories revealed that instructing participants who generate autobiographical memories to shift from a field to an observer perspective leads to decreased affective experiences (Robinson and Swanson, 1993; see also McIsaac and Eich, 2004, for the finding that observer trauma memories were experienced as less anxiety provoking than field trauma memories). These findings, possibly stemming from the fact that an observer perspective leads to the experience of greater psychological distance (e.g., between past and present selves in the case of autobiographical memories, Trope and Liberman, 2010), cast further doubt on whether objectifying situations indeed lead to the adoption of an observer's perspective.

In summary, two components of state self-objectification—adopting a third-person perspective and treating oneself as an object—have been understudied in the experimental research so far. Therefore, future research should attempt to measure these components to determine whether and how they are influenced by the induction of state self-objectification.

### Manipulations

Fredrickson et al. (1998) swimsuit-sweater paradigm, in which participants try on a swimsuit (vs. a sweater) and then look at themselves in a full-length mirror, is theorized to induce state self-objectification in participants because it increases their preoccupation with how their body looks. As Table A1 in Appendix shows, this paradigm was used in 10 different lines of research, with both male and female participants. In addition, because visual images that spotlight women's bodies and body parts “seamlessly align viewers with an implicit sexualizing gaze” (Fredrickson and Roberts, 1997, p. 176), female participants are assumed to self-objectify when exposed to sexualized depictions of women in the media (Calogero, 2011). Hence, 17 lines of research (see Table A1 in Appendix) manipulated state self-objectification in women through exposure to objectifying media, such as advertisements depicting female models in swimsuits, news items on postpartum celebrities, sexualized avatars in a virtual game, and even a film that criticizes the advertising industry's messages regarding the female body. Similarly, studies that aimed to induce state self-objectification among male participants exposed them to sexualized images of men (e.g., advertisements depicting male models in swimsuits; Rollero, 2013).

Other studies used subtler manipulations. Based on the notion that certain media contents (besides images) can objectify women (e.g., beauty advice in women's magazines, which conveys that being physically attractive should be a central life goal for women; Wolf, 1991), three lines of research (see Table A1 in Appendix) induced state self-objectification through exposure to scrambled sentences containing objectifying words such as “slender” and “beauty.” Also, based on the notion that many social contexts in women's everyday lives encourage women to think about their looks (Calogero, 2011), several studies elicited state self-objectification by exposing women to objectifying environments, such as by having scales and mirrors in the lab in which the experiment was conducted (Tiggemann and Boundy, 2008) or having participants listen to “fat talk,” where a confederate complains about her own body (Gapinski et al., 2003).

Other manipulations simulated the daily social interactions in which women experience objectification. Objectification theory posits that the anticipation of being evaluated as a sexual object by men makes women, who internalized the male gaze, preoccupied with their appearance prior to such evaluations (Calogero, 2011). Accordingly, several studies manipulated state self-objectification by leading female participants to believe they would be interacting with a man (thus creating the anticipation of “a male gaze”; Calogero, 2004), asking them to recall episodes in which they had been evaluated in a sexualized way (Calogero, 2013; Loughnan et al., 2017), and having them photographed by a male experimenter who took pictures of their body (Gay and Castano, 2010; Guizzo and Cadinu, 2016) or “checked out” by a trained male experimenter (Gervais et al., 2011)^1^.

Further, because self-objectification means being conscious of the fact that one's body is being looked at and evaluated (Fredrickson and Roberts, 1997), one study manipulated state self-objectification by instructing participants to describe their body from an observer's point of view (Register et al., 2015). Another study had male and female participants' bodies videotaped from the neck down, with participants knowing that another person (either a man or a woman) would watch the videotape later (Saguy et al., 2010). Other studies (Fea and Brannon, 2006; Tiggemann and Boundy, 2008; Kahalon et al., 2018a) manipulated state self-objectification by complimenting female participants on their appearance.

As the description above implies, all the manipulations that have been used in the literature build on objectification theory's definition of self-objectification and/or the ways through which it is enacted in women's lives. Moreover, almost all of the studies that manipulated state-self objectification used the measures described earlier as manipulation checks to confirm that it was successfully induced. Nevertheless, these manipulations are highly diverse, e.g., in terms of whether or not they involve a direct upward comparison (as in the case of exposure to images of models), or objectification by another person (vs. purely self-objectification). Given this diversity, it is perhaps not surprising that their effects—which we review in the next section—are somewhat mixed.

### The effects of state self-objectification on women

As mentioned above, when laying out objectification theory, Fredrickson and Roberts (1997) specified four direct outcomes of state self-objectification: increased body shame and anxiety (of two types—appearance and safety), and reduced experiences of flow (i.e., peak motivational states) and awareness of internal bodily states. However, the experimental literature examining the effects of this state has departed from the theory's original formulation, neglecting some of the outcomes specified by objectification theory and exploring other outcomes instead. In the following, we review the effects of state self-objectification reported in this literature on affective outcomes, behavioral and motivational outcomes, cognitive performance, and physiological outcomes.

#### Affective outcomes

A key affective response (observed in 10 different lines of research) to state self-objectification among women is body shame—the negative feeling resulting from awareness that one's body fails to meet the ideal standards set by society (and internalized by the self). Body shame, in turn, was found to predict restrained eating (Fredrickson et al., 1998), lingering body-related thoughts (Quinn et al., 2006a), and intentions to have cosmetic surgery (Calogero et al., 2014).

State self-objectification was also found to decrease women's social self-esteem (e.g., self-judgments of their social skills; Rollero, 2013), and increase body guilt (remorse over how one's body looks and desire for reparative action to “fix” it; e.g., Calogero and Pina, 2011), state anxiety (Gapinski et al., 2003), appearance-based anxiety (anxiety about being negatively evaluated by others because of one's overall appearance; e.g., Calogero, 2004), and dissatisfaction with one's body (e.g., Aubrey et al., 2009). Several other studies found state self-objectification to decrease women's positive affect (e.g., Rollero, 2013) and increase their negative affect (e.g., Tiggemann and Andrew, 2012).

However, when state self-objectification was manipulated through appearance compliments, it led to improved mood either generally (Tiggemann and Boundy, 2008) or specifically among women with high trait self-objectification (Fea and Brannon, 2006; Kahalon et al., 2018a). Similarly, watching a film critical of the advertising industry's excessive emphasis on the thinness ideal improved women's mood (Choma et al., 2007). Finally, receiving appearance compliments while being exposed to death primes (which increases people's wish to validate their cultural worldview and view of themselves; Greenberg et al., 2008) boosted the self-esteem of women with high trait self-objectification (Goldenberg et al., 2011). Thus, at least for some women (those high on self-objectification) and under certain conditions, state self-objectification has positive affective consequences. For example, a woman who decides to post her picture in swimsuit on Facebook may be in a state of self-objectification (e.g., thinking about her physical appearance) yet feel proud and happy about it (for a discussion of women's enjoyment of their sexual objectification, see Liss et al., 2011).

#### Behavioral, motivational, and attitudinal outcomes

Beyond the effects mediated through increased body shame (see *affective outcomes*), state self-objectification was shown to increase women's drive for thinness (Register et al., 2015), and lead them to perceive the physical aspects of sex (e.g., feeling the partner's genitals) as less appealing (Roberts and Gettman, 2004), talk less in cross-gender interactions (Saguy et al., 2010), show greater endorsement of rape myths (Fox et al., 2014), and justify the existing gender system, which in turn reduced their intentions to engage in collective action for gender equality (Calogero, 2013). Thus, state self-objectification leads women to “narrow their presence” (Saguy et al., 2010) and endorse ideologies and behaviors that perpetuate traditional, unequal gender roles.

#### Cognitive performance

Several studies (Fredrickson et al., 1998; Hebl et al., 2004; Gay and Castano, 2010; Gervais et al., 2011; Kahalon et al., 2018a) found that state self-objectification impaired women's math performance. For women with high trait self-objectification, state self-objectification also impaired performance on a Letter Number Sequencing (LNS) task, which tests working memory capacity (Gay and Castano, 2010). For women with high internalization of beauty ideals, state self-objectification impaired performance in a Sustained Attention to Response Task (SART) that tests the ability to sustain attention on repetitive stimuli and increased feelings of imbalance between the participant's skills and the task's demands (Guizzo and Cadinu, 2016). Finally, state self-objectification increased women's response latencies on a modified Stroop task (Quinn et al., 2006b); however, as explained below, the analysis used in that study raises concerns about its conclusions.

In contrast, Gapinski et al. (2003) did not find evidence for impaired math performance under objectifying conditions. Similarly, Tiggemann and Boundy (2008) found no effect of state self-objectification on women's cognitive performance, as evaluated through performance on tests of logical reasoning and spatial orientation. All in all, there is quite convincing evidence that state self-objectification does impair women's cognitive performance, yet the evidence is somewhat mixed pertaining to whether or not this impairment is moderated by women's trait self-objectification and/or internalization of beauty ideals.

#### Physiological outcomes

Green et al. (2012) found that, regardless of their level of trait self-objectification, women had a lower mean heart rate when trying on a swimsuit as compared to a tracksuit. This effect is indicative of a prolonged “orienting response” (heightened cognitive processing of a given stimulus; Graham and Clifton, 1966). According to Green et al. (2012), this prolonged orienting response may explain the decreased cognitive performance under objectifying conditions (reported in other studies), because orienting one's attentional resources to a given stimulus reduces the ability to process competing stimuli.

A subsequent study by Green et al. (2014) extended their original study by including: (a) male participants (who tried on Speedos in the swimsuit condition), and (b) two control conditions—observing nature images and trying on perfume—which involved no objectification (the rationale being that trying on a tracksuit does induce a certain degree of state self-objectification). Replicating Green et al. 's (2012) results, the pattern of women's heart rates indicated a prolonged orienting response in the high-objectification (swimsuit) as compared to the low-objectification (unisex tracksuit) condition. However, this effect was found among men as well, indicating that they, too, had a prolonged orienting response in the swimsuit condition. Moreover, in the objectifying (swimsuit/tracksuit) conditions, as compared to the non-objectifying (perfume/nature images) conditions, both women and men showed accelerated heart rate, indicative of greater cardiac autonomic stress. This effect, however, was stronger among women, suggesting that under objectifying conditions women's stress was greater than men's.

#### Summary and future research

There is consistent evidence that state self-objectification in women increases behavior that aligns with the traditional feminine role (i.e., narrowing their presence in cross-gender interactions and accepting the existing gender system) and has negative consequences in terms of affective response (with some exceptions, such as when receiving appearance compliments) and cognitive performance (at least under certain circumstances and/or for some women). In terms of physiological outcomes, there is initial evidence for stress and diversion of attention under objectifying conditions. Surprisingly, despite the prominence of these outcomes in objectification theory (Fredrickson and Roberts, 1997), there is no reported evidence for experimentally induced effects on awareness of internal bodily states and/or experience of safety anxiety (fear of “the potential for sexually motivated bodily harm”; p. 183). As for disruption to flow experiences, it was examined in only one study (Guizzo and Cadinu, 2016), which used a repetitive, boring task and focused on a single aspect of this construct (retrospective experience of balance between skills and task's demands; other aspects of flow were not influenced by the objectifying condition). Future research should therefore address these lacunae (e.g., by examining disruption to flow in challenging, enjoyable tasks; Csikszentmihalyi, 1990).

Future research is also required to increase knowledge on state self-objectification among men. Existing experimental research (see Table A1 in Appendix) produced somewhat inconsistent results. Several studies found no effect of objectifying conditions on men's body shame and restrained eating (Fredrickson et al., 1998), negative affect and appeal of physical sex (Roberts and Gettman, 2004), body surveillance and dissatisfaction (Gervais et al., 2011), body-related thoughts (Quinn et al., 2006a), depressive feelings (Register et al., 2015), perception of one's muscularity (Johnson et al., 2007), amount of talking in either same-gender or cross-gender interactions (Saguy et al., 2010), or math performance (Fredrickson et al., 1998; Gervais et al., 2011). However, in other studies, objectifying conditions increased men's body shame and impaired their math performance (Hebl et al., 2004; Kahalon et al., 2018a), decreased men's positive affect (Rollero, 2013), increased men's drive for thinness (Register et al., 2015), and led to prolonged orienting responses, increased cardiac stress reactions, decreased positive affect (Green et al., 2014), and improved mood for men high in TSO (Kahalon et al., 2018a). Studies that explored the effects on heterosexual men as compared to gay men also yielded mixed results (see Michaels et al., 2013, vs. Martins et al., 2007). Research on the effects of state self-objectification on men is interesting both because the objectification of men in Western society is on a constant increase (Rhode, 2010) and because it can shed light on the processes triggered by this state and on whether they differ among men and women.

## Psychological mechanisms through which state self-objectification exerts its effects on women: an integrative process model

Although the body of work mentioned above establishes that state self-objectification has detrimental effects on women, it has not adequately identified or distinguished between the psychological mechanisms underlying these effects. The mechanism originally proposed by objectification theory is appearance monitoring, which depletes attentional resources. According to Fredrickson et al. (1998), “[s]elf-objectification leads to a form of self-consciousness characterized by vigilant monitoring of the body's outward appearance. This self-conscious appearance monitoring can disrupt an individual's stream of consciousness, and thereby limit the mental resources available for other activities” (p. 270). Although this mechanism is compatible with many of the findings reported in the literature, direct attempts to test it have been fairly limited (e.g., only six experiments that manipulated state self-objectification in women actually measured appearance monitoring). Moreover, as we explain below, attempts to test and/or rule out alternative mechanisms have been insufficient, and several of the outcomes reported in the literature (e.g., support for existing gender arrangements; Calogero, 2013) cannot be accounted for by appearance monitoring *per se*.

In light of the understanding that a multifaceted phenomenon is likely to reflect several processes rather than a single one, we suggest a model, illustrated in Figure 1, in which state self-objectification (induced through an objectifying situation, such as trying on a swimsuit or receiving an appearance compliment) can trigger four different processes. These processes reflect the social-psychological mechanisms through which a state of self-objectification can affect the various outcomes reported in the literature. In addition to appearance monitoring^2^, the four mechanisms include: the experience of discrepancy from appearance standards, stereotype threat, and activation (i.e., priming) of the “sex object” schema. We now discuss the theoretical justifications and empirical evidence for each of these mechanisms.

### Appearance monitoring

Appearance monitoring, sometimes termed “self-surveillance,” “body surveillance,” and “body monitoring” refers to the repeated monitoring of, and diversion of attention to, one's outward appearance (note that we prefer the term referring to “appearance” rather than “body,” because it has a broader meaning, such as monitoring one's makeup, clothes, or hair). According to objectification theory, this process accounts for the four negative psychological consequences that women experience when induced with state self-objectification (increased shame and appearance/safety anxiety, and reduced flow and awareness of internal bodily states; Fredrickson and Roberts, 1997). Moreover, because it diverts attention from the task at hand, appearance monitoring is also said to lead to the impairment in cognitive performance observed among women in a state of self-objectification (Fredrickson et al., 1998).

Of the six studies mentioned above that measured appearance monitoring, four found that it increased in women following the induction of state self-objectification (Calogero and Pina, 2011; and Tiggemann and Andrew, 2012; Ford et al., 2015; Hopper and Aubrey, 2016). Further support for this mechanism is provided by the results of studies that found the effects of state self-objectification to be stronger among women high on trait self-objectification—namely, women who are chronically preoccupied with their appearance (note that being high in trait self-objectification means diverting much attention to one's appearance, not necessarily feeling dissatisfied with it; Frederick et al., 2007; Calogero, 2011). To illustrate, the impairment to cognitive performance after having their bodies filmed by a male confederate (Gay and Castano, 2010) was greater among women high (vs. low) in trait self-objectification. The fact that women's chronic preoccupation with their appearance influences their vulnerability to state self-objectification is consistent with appearance monitoring as a mechanism.

Beyond this evidence, two studies attempted to provide direct evidence for women's attentional deficits due to the diversion of attention to monitor their bodies in a state of self-objectification. Quinn et al. (2006b) demonstrated that women were slower to respond to a Stroop task when trying on a swimsuit vs. a sweater. However, instead of interference scores (i.e., the difference in reaction times for incongruent vs. congruent trials; Kane and Engle, 2003), Quinn et al. reported participants' overall response times, showing that participants were *generally* slower to respond in the swimsuit condition. Whereas greater interference scores indicate that attention failed to inhibit the irrelevant aspect of the incongruent stimulus, overall response times indicate participants' general speed, which is not diagnostic of any specific attentional deficits (actually, interference scores are calculated in order to *partial out* participants' overall speed; Kane and Engle, 2003). Hence, the analysis of the study's results renders them uninterpretable. A recent experiment by Guizzo and Cadinu (2016), however, provided more convincing evidence for attention deficits, demonstrating that when a male (vs. a female) experimenter took photographs of their bodies, female participants high in internalization of beauty standards performed worse on the SART (in which participants are required to withhold responses to infrequent stimuli while responding to frequent ones, indicating sustained attention; Robertson et al., 1997).

Despite the support for this mechanism, appearance monitoring cannot account for two types of evidence. First, Gervais et al. (2011) found that being “checked out” by a male experimenter impaired women's math performance, even though it did not lead to more appearance monitoring than in the non-objectifying control condition. This suggests that a mechanism other than appearance monitoring was responsible for the observed impairment. Second, several studies found that like women, men, too, monitor their appearance when objectified. Green et al. (2014) found that under objectifying conditions, both men and women diverted attentional resources to their bodies, as indicated by prolonged orienting responses. Other studies, which used the TST, indicated that objectifying conditions led men and women alike to define themselves in terms of their body attributes. For example, Fredrickson et al. (1998) found that both men and women generated more appearance-related statements in the swimsuit condition, with no gender by condition interaction (see also Roberts and Gettman, 2004; Quinn et al., 2006a). The fact that under state self-objectification both men and women divert their attention to their appearance but that this state produces gender differences in other outcomes (such as math performance) suggests that a process other than appearance monitoring accounts for the observed differences.

To summarize our argument, there is evidence that appearance monitoring increases under state self-objectification and is responsible for some of the effects reported in the literature (e.g., the ones moderated by trait self-objectification). However, appearance monitoring cannot account for all of the findings (especially the findings pertaining to gender differences), suggesting that other mechanisms must be at work as well. Building on and integrating diverse social psychological literatures, in the following we identify three additional processes that might account for the effects of self-objectification reported in the experimental research on state self-objectification.

### Discrepancy from appearance standards

Consistent with the argument spelled out in the previous section, Quinn et al. (2011) noted that manipulations of state self-objectification induce “objective self-awareness” (p. 129) in both women and men, in which they focus their attention on their appearance. Therefore, the mechanism leading to gender differences in outcomes such as restrained eating or impaired cognitive performance cannot be appearance monitoring *per se*. To account for these gender differences, Quinn et al. (2011) combined objectification theory's suggestion that when women divert their attention to their body, they also engage in comparing it to the mythic ideal body (Fredrickson and Roberts, 1997) with insights from the literature on self-regulation (Carver and Scheier, 1998).

Specifically, the theory of objective self-awareness (Wicklund, 1975) tells us that the initial reaction to events that force attention inward (such as reflections of the self), as opposed to events that pull attention outward (such as distracting stimuli outside the self), is self-evaluation. Applying this theory to the contexts examined in the objectification literature, Quinn et al. (2011) argued that objective self-awareness automatically activates internalized appearance standards. Because women have stringent beauty standards, they experience considerable discrepancies from the activated standards. This experience (i.e., cognitive appraisal of discrepancies) prompts a host of negative self-conscious emotions, such as body shame and appearance anxiety. These emotions lead to self-regulatory attempts to reduce the existing discrepancies (e.g., by restraining one's eating or considering undergoing cosmetic surgery). The engagement in self-regulation interrupts women's experience of flow states and interferes with their cognitive performance. Men, in contrast, find fewer discrepancies between the internalized standards and their own appearance. They are therefore quicker to exit “the self-regulatory loop” (p. 131) of efforts to eliminate these discrepancies and hence typically do not suffer from interruption to flow states and cognitive performance under objectifying conditions. Thus, Quinn et al. (2011) identify the experience of discrepancy from appearance standards as a critical psychological process (i.e., mechanism) set in motion by state self-objectification in women.

Departing slightly from Quinn et al. 's (2011) theorizing, our proposed framework conceptualizes appearance monitoring and the experience of discrepancy from ideal appearance standards as two distinct processes. The purpose of this distinction is to stress that diversion of attention to one's appearance is a necessary, but not a sufficient condition to the activation of, and consequent experience of discrepancy from internalized appearance standards. A corresponding distinction was made in the literature on trait self-objectification, which denotes preoccupation with one's appearance but “without a judgmental or evaluative component” (Calogero, 2011, p. 24), and which may or may not be associated with body dissatisfaction. In the case of state self-objectification, it is possible for women to monitor their appearance (e.g., when fixing their hair before a romantic date) without experiencing discrepancy from internalized ideal standards. Unfortunately, however, it is often the case that women's monitoring of their appearance results in experiencing this discrepancy.

### Schema activation (priming)

Priming denotes the automatic activation of knowledge structures (Bargh, 2006). Experimental manipulations of priming typically involve exposure to pictures, objects, or words that are related to a certain construct, thus temporarily increasing its accessibility (Bargh, 2006). Earlier theorizing (e.g., Bargh et al., 1996) suggested a simple prime-to-behavior sequence, such that the priming of a particular social category generally leads to responses congruent with the stereotypical traits ascribed to it. Later research, however, offered a more nuanced picture, suggesting that priming influences behavior by making certain contents of one's self-concept temporarily active (Wheeler et al., 2014). It can therefore lead not only to prime-congruent behavior (assimilation effects) but also to prime-incongruent behavior (contrast effects). To illustrate the latter, when focused on their young student identity, participants distanced themselves from the activated stereotypes of the elderly (e.g., they responded to words such as “forgetful” more slowly; Spears et al., 2004).

We suggest that existing manipulations of state self-objectification may prime participants with the concept of “sex objects,” which is a traditionally feminine role (Dworkin, 1974). While this prime should not influence men (who might even distance themselves from the activated category; Spears et al., 2004), it should increase the accessibility of contents in women's self-concepts that are associated with the sex object schema, leading them to align their perceptions, motivations, and behavior with those expected from sex objects. To illustrate, exposing women to words like “sexiness” within scrambled sentences may lead to behavior that is consistent with the role of a sex object, such as engagement in beauty practices (Calogero et al., 2014).

Because research on state self-objectification has not considered schema activation as a mechanism, there is currently no empirical evidence of the kind typically reported in the priming literature (e.g., response times in a lexical decision task; Neely, 1977) in support of it. However, several of the reported effects in objectification studies are best accounted for by schema activation. Specifically, Calogero (2013) found that under objectifying conditions, women were less supportive of action to reduce gender inequality. This effect cannot be accounted for by appearance monitoring or experience of discrepancy from appearance standards—but is readily explained by schema activation. In fact, even though the term “schema activation” is not explicitly used by Calogero, the argument that women's state self-objectification “leads them to comply with traditional gender roles (e.g., that of a sex object)” (Calogero, 2013, p. 317) is consistent with this mechanism. In yet another example, the suggestion that “women talk less when objectified because they attempt to align their behavior with what they assume is expected of them as sexual objects” (Saguy et al., 2010, p. 182) is consistent with schema activation as a mechanism.

### Stereotype threat

Stereotype threat denotes stigmatized group members' concern regarding the possibility of confirming the negative stereotype about their group (Steele, 1997; see also Shapiro and Neuberg, 2007). This concern causes stress that undermines stigmatized group members' actual performance in stigmatized domains. Importantly, the experience of stereotype threat increases in response to situational cues that increase the salience of one's group membership (Schmader et al., 2008). For example, women's math performance was impaired due to stereotype threat when they were tested in a room along with two other men, as opposed to two other women or one man and one woman (Inzlicht and Ben-Zeev, 2000).

We suggest that the manipulations used to induce state self-objectification in female participants may increase the salience of their group membership—namely, remind them that they are women. When the outcome variable in the experiment is performance in a stigmatized domain, these reminders can increase stereotype threat. Consequently, women's performance might be impaired, as was found, for example, for mental rotation tasks (Gapinski et al., 2003) and math (e.g., Gervais et al., 2011, who indeed acknowledged that “[t]o the degree that the objectifying gaze arouses stereotype threat, math performance may decrease” [p. 5]).

Fredrickson et al. (1998) attempted to rule out stereotype threat as an alternative explanation of the effect of the swimsuit condition on women's math performance by arguing that it is “improbable that women in the sweater condition were unaware of the gender stereotype regarding math” and hence “all of our female participants—those wearing sweaters and those wearing swimsuits—were performing under stereotype threat conditions” (p. 280). However, more recent theorizing (Schmader et al., 2008) suggests that stereotype threat effects do not take the form of “all or none:” Conditions that increase the saliency of women's gender induce higher levels of stereotype threat and subsequent performance impairment. In light of these more recent data, Fredrickson et al.'s (1998) argument does not convincingly rule out stereotype threat as a mechanism.

One study that seems to rule out stereotype threat is Hebl et al. (2004), which found that highly objectifying conditions (wearing Speedos) led to impaired math performance even among men. If the effect of state self-objectification was driven by stereotype threat, it should have emerged only among women. However, Hebl et al. examined participants of various ethnicities (African American, Hispanic, Caucasian, and Asian American). It is possible that looking at themselves in the mirror reminded African American and Hispanic men of their ethnic/racial identity, which increased their experience of stereotype threat and consequently impaired cognitive performance. Although Hebl et al. did not report the results of the condition × gender × ethnicity/race interaction, the observed performance decrements (31 and 20% among objectified Hispanic and African American men, vs. 7% among objectified white men) are consistent with this possibility. Therefore, even Hebl et al.'s findings cannot rule out stereotype threat as a mechanism.

Still, one could argue that studies that found impaired cognitive performance on non-stigmatized tasks such as the Gestalt test (identifying partially degraded images of various objects; Gapinski et al., 2003) or the LNS test (Gay and Castano, 2010) rule out stereotype threat as a mechanism (based on the rationale that stereotype threat impairs performance only in stigmatized domains; see Quinn et al., 2006b). However, current theorizing claims that stereotype threat can lead to general rather than domain specific impairments to cognitive performance. For example, women who were told that they were going to take a math test (vs. a verbal test) later on performed worse on non-stigmatized tasks such as a Stroop test (Inzlicht et al., 2006). In other words, simply expecting to be tested in a stigmatized domain at some later point in time leads to the experience of stereotype threat, which “weakens the ability to control and regulate one's behaviors in domains unrelated to the stigma” (Inzlicht et al., 2006, p. 262). It is possible that participants in the Gapinski et al. (2003) or Gay and Castano (2010; Study 1) experiments experienced stereotype threat because they knew they were about to take a math test later. This experience of stereotype threat (resulting from the expectation of a math test) intensified in the objectifying conditions, leading to performance decrements even in the non-stigmatized tasks included in these studies.

Finally, the performance deficits observed by Guizzo and Cadinu (2016) cannot be explained by stereotype threat, because the SART (measuring sustained attention; Robertson et al., 1997) was in a non-stigmatized domain and participants were not about to complete a test in a stigmatized domain. However, because all participants in the study were women, it is unknown whether male participants would show similar attention deficits—a possibility that seems plausible given Green et al. 's (2014) findings that both men and women have prolonged orienting responses when objectified. In other words, Guizzo and Cadinu's (2016) findings cannot rule out the possibility that: (a) both men and women suffer from attentional deficits under objectifying conditions (hence, both genders would show performance deficits in tasks such as SART or Stroop when objectified), but (b) when the task involves being tested in a stigmatized domain, such as math, objectified women *additionally* suffer from stereotype threat, which is responsible for their performance deficits compared to objectified men.

To summarize, the situations that induce women with state-self objectification typically increase the saliency of their gender, which increases their vulnerability to stereotype threat (Schmader et al., 2008). Even though several studies have argued or otherwise seem at first glance to rule out stereotype threat as a mechanism, a closer inspection of their results reveals that there are insufficient grounds for doing so. Thus, some of reported effects of state self-objectification on women's cognitive performance (i.e., the effects observed in studies that included tests in stigmatized domains) might stem from stereotype threat.

### Summary

Figure 1 presents our integrative process model. According to the model, a state of self-objectification, introduced by an objectifying situation (e.g., entering a room that contains scales, full-length mirrors, and a display of fashion magazine covers; Tiggemann and Boundy, 2008), may trigger four processes: appearance monitoring, experience of discrepancy from appearance standards, activation of the sex object schema, and the experience of stereotype threat. Whereas some objectifying situations trigger all four processes, others may trigger only one or some of them. For example, receiving an appearance compliment (Fea and Brannon, 2006) may increase a woman's appearance monitoring without leading her to experience discrepancy from appearance standards. And stereotype threat is likely to be experienced by objectified female participants when they are required to perform a task in a stigmatized domain (e.g., complete a math test; Fredrickson et al., 1998) but not when the experimental design does not include task performance assessment.

Notably, we argue that three of these processes can occur independently of the others, yet the experience of discrepancy from appearance standards is preceded by appearance monitoring (i.e., diversion of attention to one's looks; see Quinn et al., 2011). Also, priming women with the sex object schema is one way through which the saliency of their gender may be heightened; therefore, activating the sex object schema may increase stereotype threat (see Marx, 2012, for a discussion of how priming, which is a “cold” cognitive process, might increase stereotype threat, which is a “hot” motivational process). The above-mentioned links between the first two processes (appearance monitoring and experience of discrepancy from appearance standards) and the latter two processes (schema activating and stereotype threat) are represented by the dashed lines in Figure 1.

As noted above, our model views the four processes as mechanisms leading to the various outcomes reported in the experimental literature on state self-objectification in women. Each process is related to a different set of outcomes. Appearance monitoring depletes attentional resources and therefore leads to impaired cognitive performance, which is represented by the respective arrow in Figure 1. In itself, appearance monitoring does not lead to negative affective changes such as body shame or increased motivation to improve appearance; these occur only when a woman feels that she falls short of the conventional beauty standards.

Specifically, the experience of discrepancy from appearance standards leads to a host of affective changes, such as increased body shame and to increased motivation to improve one's appearance through restrained eating, intentions to undergo plastic surgery, and so forth (see Quinn et al., 2011). Also, because failing to meet conventional standards leads to the experience of anxiety (worry and nervousness accompanied by physiological arousal; Weiner and Craighead, 2010), and anxiety interferes with executive functions (Eysenck et al., 2007), the experience of discrepancy from appearance standards should lead to impaired cognitive performance. These paths are represented by three respective arrows in Figure 1.^3^

Activation of the sex object schema also leads to three sets of outcomes: a heightened motivation to improve one's appearance, impaired cognitive performance (see Jamieson and Harkins, 2012, for priming effects on women's math performance), and stereotype consistent behaviors (e.g., exhibiting “narrowed presence,” Saguy et al., 2010; or accepting ideologies that justify women's subordination, such as rape myths; Fox et al., 2014). These outcomes align with the excessive engagement in beauty practices (Wolf, 1991), submissiveness (“Objects don't object”; Calogero, 2013, p. 312), and lack of competence (Heflick and Goldenberg, 2009) that is expected from sex objects. As a “cold” and automatic cognitive process of content activation (Marx, 2012), priming the sex object schema is not theorized to lead to affective changes. The proposed three paths are represented by respective arrows in Figure 1.

Finally, stereotype threat leads to impaired cognitive performance (Steele, 1997; Schmader et al., 2008) as well as to negative affective responses such as anxiety (e.g., Spencer et al., 1999). Yet there is no theoretical or empirical basis to expect stereotype threat to be related to the motivation to improve one's appearance or to stereotype consistent behavior such as submissiveness (e.g., narrowed presence) and support for patriarchal arrangements. In particular, stereotype threat is a motivational process in which one's wish to refute the negative stereotype about one's group triggers stress, monitoring, and suppression processes that impair the executive functions necessary for successful task performance (Schmader et al., 2008). These processes are not expected to influence outcomes such as amount of talking in cross-gender interactions (Saguy et al., 2010), justification of the existing gender system justification (Calogero, 2013) or acceptance of rape myths (Fox et al., 2014), which are best accounted for by schema activation. The proposed two paths are represented by respective arrows in Figure 1.

Before we proceed to the final section, where we outline several suggestions on how future research could test the mechanisms put forward in Figure 1, two clarifications are in order. First, our model refers only to existing findings within the experimental literature. Therefore, it does not include outcomes such as safety anxiety and awareness to internal states (see Fredrickson and Roberts, 1997), for which there is currently no empirical evidence. Second, the model in Figure 1 seeks to explain the mechanisms triggered by state self-objectification in women. The experience of state of self-objectification in men (e.g., through exposure to idealizing media images; Michaels et al., 2013) can trigger appearance monitoring and experience of discrepancy from appearance standards. However, state self-objectification is unlikely to affect men's cognitive performance (in the traditionally masculine domains examined in the objectification literature, such as logical reasoning or math) through the induction of stereotype threat, because men's gender identity is not stigmatized in these domains (albeit men might experience stereotype threat in traditionally feminine domain; Kahalon et al., 2018b). Hence, increasing the salience of men's gender identity should not result in decrements to their cognitive performance due to stereotype threat (Schmader et al., 2008) or the priming of the sex object schema (from which men are likely to distance themselves, revealing prime-incongruent behavior; Spears et al., 2004). That some of the social psychological mechanisms triggered by state self-objectification are unique to women, whereas others are common to both genders, can explain why some studies find gender differences (e.g., Saguy et al., 2010) but others find similarities (e.g., Hebl et al., 2004).

## Future research disentangling the processes triggered by state self-objectification

The operation of the four proposed mechanisms can be empirically identified both through direct measures and by examining moderators that are uniquely associated with each of them. We now turn to explain these two strategies.

### Direct measures of the mechanisms

To establish that monitoring one's appearance (first mechanism in Figure 1) is the mechanism that drives a given effect of state self-objectification, the experimental procedure could include a measure of participants' appearance monitoring (e.g., the modified surveillance subscale of the OBCS; Breines et al., 2008). Although never conducted experimentally, it is also possible to measure actual appearance monitoring behavior (e.g., looking in the mirror). Once these measures are included in the experimental procedure, tests of indirect effects could be used to detect whether participants' appearance monitoring mediates the effects of state self-objectification on the outcome variable of interest (e.g., performance in a logical reasoning test; Tiggemann and Boundy, 2008).

The second mechanism in Figure 1 is experience of discrepancy from appearance standards, which can be assessed by Stunkard's figure rating scale (Stunkard et al., 1983). The scale presents a series of schematic figures and asks participants to select the one that represents their own body and the one that they view as the ideal body; this allows assessment of the discrepancy between the two. To establish that a given effect of state self-objectification is driven by the experience of discrepancy from appearance standards, the procedure could include Stunkard's figure rating scale and test whether it serves as a mediator.

To examine activation of the sex object schema as a mechanism (the third in Figure 1), research could use common techniques to detect priming effects. These include word-stem or word-fragment completion tasks (completing a word in which some letters are missing; Tulving et al., 1982) or a lexical decision task (determining whether a given string of letters constitutes an existing word; Neely, 1977). If participants are more likely to complete stems/fragments with objectifying words (e.g., completing TH_N as “thin” rather than “then” or “than”) or are quicker to identify words like “sexy,” it may indicate the activation of the sex object concept. Again, analysis could determine whether such activation patterns mediate the effects of state self-objectification on the outcome variable of interest (e.g., support for the existing gender arrangements; Calogero, 2013).

Finally, to examine stereotype threat as a mechanism (influencing outcomes such as math performance, Fredrickson et al., 1998; or negative feelings, Gapinski et al., 2003), it is possible to include a measure of threat appraisal. This measure, consisting of items such as “I worry that my ability to perform well on math tests is affected by my gender,” was used in previous research to detect stereotype threat (Marx and Goff, 2005). Finding that threat appraisals mediate the effect of state self-objectification on performance in a stigmatized domain or negative feelings would establish that stereotype threat was responsible for the observed effect.

Another strategy to establish stereotype threat as a mechanism is using a blockage design (Mackinnon, 2008), as is typically done in the stereotype literature. To illustrate, Spencer et al. (1999) established that stereotype threat occurred by demonstrating that when the stereotype regarding their inferior math ability was explicitly refuted (thus blocking the proposed mediating variable), women's math performance was no longer worse than that of men's. In a similar vein, if the negative effect of a given manipulation of state self-objectification (e.g., trying on a swimsuit) on women's math performance disappears once participants are told that the math test they are about to take produces no gender differences, this would demonstrate that this effect was driven by stereotype threat. If the effect persists despite such explicit refutation, this would indicate that it was triggered by another mechanism (e.g., depletion of attentional resources due to appearance monitoring).

### Moderators uniquely associated with each mechanism

Moderator variables can also shed light on the operation of the four mechanisms. As mentioned above, the finding that women who are high in trait self-objectification; i.e., chronically preoccupied with their appearance (regardless of their satisfaction with it) are more vulnerable to the effects of state self-objectification is consistent with appearance monitoring as a mechanism. Based on similar reasoning, finding that participants' internalization of cultural appearance standards (Heinberg et al., 1995) moderates the effects of objectifying conditions would be consistent with discrepancy from appearance standards as a mechanism.

Level of self-monitoring (Snyder, 1974) is a moderator that is uniquely associated with priming. According to DeMarree et al. (2005), primes increase prime-consistent behavior when the activated construct is misattributed to the self—namely, when people confuse accessible mental contents with their own traits. People who are low in self-monitoring—that is, who look inward and rely more heavily on their own traits, feelings, and beliefs to guide their actions (Snyder, 1974) are more likely to be affected by primes. This is because they mistakenly perceive the subtle activation of traits and constructs as conveying information about their self-characteristics or subjective feelings (DeMarree et al., 2005). Hence, finding that an effect of state self-objectification is stronger among women low in self-monitoring would be consistent with priming as a mechanism.

Finally, domain identification—the extent to which the stigmatized domain is perceived by a woman as important, feasible, and rewarding (Smith and White, 2001) is a moderator that is uniquely associated with stereotype threat. For instance, women with high identification with the math domain are more vulnerable to stereotype threat than women with low math identification (Keller, 2007). A second moderator that is uniquely associated with stereotype threat is stigma consciousness—the extent to which a woman is chronically self-conscious of her stigmatized status (Brown and Pinel, 2003). Finding that the effect of objectifying conditions is moderated by either domain identification or stigma consciousness would be consistent with stereotype threat as a mechanism.

### Moderation by demographic variables

As is typically the case in mainstream social psychological research (Shields, 2008), the populations investigated have not been diverse in terms of demographic variables (see Table A1 in Appendix). However, there are critical differences between women who belong to different social categories: lesbians are more satisfied with their body than heterosexual women are (Morrison et al., 2004), black women prefer a curvier body than white women do (Overstreet et al., 2010), women in steady, long-term relationships place less importance on their appearance than women who are currently looking for a partner do (Sanchez and Broccoli, 2008), older women exhibit lower levels of habitual body monitoring and appearance anxiety than younger women do (Tiggemann and Lynch, 2001), and women from Western societies (Australia, Italy, UK, and the US) report higher levels of trait self-objectification compared to women from Eastern cultures (India, Japan, and Pakistan; Loughnan et al., 2015).

Exploring whether these variables moderate the effects of state self-objectification on outcomes of interest can shed light on the mechanisms driving these effects. For instance, finding that the effect of the swimsuit manipulation on math performance is stronger for heterosexual women than for lesbians would be consistent with discrepancy from appearance standards as a mechanism but not with stereotype threat. In yet another example, because motherhood is negatively associated with sexiness (the Madonna-whore dichotomy; Bareket et al., 2018), mothers may be less prone to associate the category of “sex object” with their self-concepts. Hence, finding that the effect of objectifying conditions on a particular outcome variable (e.g., amount of talking in cross-gender interactions) is weaker among mothers than in non-mothers would be consistent with schema activation as a mechanism.

On a broader level, exploring the universality of state self-objectification effects in women can provide critical insights into the nature of this phenomenon. According to evolutionary psychology, both men and women engage in assessing women's beauty to increase their reproductive fitness (Sugiyama, 2005). Men do so because features associated with women's beauty indicate their “mate value” (potential to promote the reproductive success of the man mating with them); women do so because assessing their own attractiveness vis-à-vis other women guides their decisions about whether to compete over potential male mates. If so, the societal objectification of women should be a universal phenomenon (see Gottschall et al., 2008). By contrast, feminist theorizing (e.g., Wolf, 1991) argues that the objectification of women, and consequent self-objectification phenomena (such as eating disorders), have substantially increased following the second wave of feminism—representing a backlash to women's empowerment in Western societies. If so, in more traditional societies, where more blatant mechanisms are used to police and limit women's power (e.g., giving them less legal rights than men)—women should be less susceptible to the negative effects of state self-objectification. Cross cultural research can shed light on this theoretical dispute, revealing whether the negative effects of self-objectification are inherent to being a woman, or if they represent a contemporary western phenomenon that has historical and cultural boundaries.

## Conclusion

Experimental research on state self-objectification has been growing rapidly over the last 20 years. The present paper provides an organizing framework for this body of research by addressing three issues. First, the measures and manipulations of state self-objectification in the existing experimental research focused on one component of this construct: heightened preoccupation with one's physical appearance. Two additional components—adopting a third-person's perspective and reducing oneself to the status of an object—have been relatively understudied and require additional empirical testing. Second, in its original formulation, objectification theory (Fredrickson and Roberts, 1997) specified four key outcomes of self-objectification: body shame, appearance and safety anxiety, reduced flow experiences, and less awareness of internal bodily states. Whereas body shame and appearance anxiety have been thoroughly researched, the other outcomes need further investigation. Identifying these lacunae is an important contribution of the present paper.

Third, and most importantly, our integrative process model suggests that the induction of state self-objectification triggers four distinct psychological processes in women: diverting attention to appearance monitoring, experience of discrepancy from appearance standards, stereotype threat, and activation of the “sex object” schema. These conceptually distinct mechanisms have not been sufficiently differentiated from each other empirically, which can be accomplished both by directly measuring these mechanisms and by testing the moderators uniquely associated with each of them. Organizing the literature in terms of the proposed model has theoretical and practical importance.

Theoretically, the body of work on state self-objectification can and should be better integrated with other relevant social psychological work, such as theorizing on subtle, even seemingly benevolent forms of sexism (see Calogero and Jost, 2011, for a stepping stone in this direction). However, the absence of a clear understanding of how state self-objectification influences women impedes such theoretical progress. The present analysis can facilitate these theoretical advances by introducing a holistic model that adds new, theory-based links that have not been empirically tested so far to the links already established in the objectification literature (such as the links between state self-objectification, experience of discrepancy from appearance standards, and body shame; see Quinn et al., 2011). This integrative model may serve as a roadmap that directs future research in a systematic manner.

At the practical level, developing effective psychological interventions to prevent unwarranted outcomes must begin by clearly identifying the mechanisms responsible for these outcomes (Walton, 2014). We hope that our model will contribute to this goal and help practitioners to develop interventions to buffer the detrimental effects of state self-objectification. In a society that is constantly increasing the emphasis on women's appearance (Zurbriggen et al., 2007; Rhode, 2010), this is an urgent goal.

## Author contributions

RK analyzed the existing literature and conceptualized the integrative process model presented in the paper, drafted the first version of the paper and participated in the revision process, approved the paper's final version, and agreed to be accountable for all aspects of the work. NS substantially contributed to the conceptualization of the integrative process model presented in the paper, thoroughly participated in the revision process, approved the paper's final version, and agreed to be accountable for all aspects of the work. JB substantially contributed to the conceptualization of the integrative process model presented in the paper, thoroughly participated in the revision process, approved the paper's final version, and agreed to be accountable for all aspects of the work.

### Conflict of interest statement

The authors declare that the research was conducted in the absence of any commercial or financial relationships that could be construed as a potential conflict of interest.

## Footnotes

^1^Admittedly, Gervais et al. (2011) and Guizzo and Cadinu (2016) did not explicitly attempt to manipulate participants' *self*-objectification. However, we included these studies in our review, because: (a) their theoretical rationale is built on objectification theory, in which the objectifying male gaze is hypothesized to exert its effects on women through the process of self-objectification, and (b) their manipulations bear close resemblance to that used by Gay and Castano (2010), who did attempt to manipulate *self*-objectification.

^2^Admittedly, there is disagreement in the literature about the extent to which self-objectification and appearance monitoring are the same construct (for a discussion of this issue see Calogero, 2011; Lindner and Tantleff-Dunn, 2017). Consistent with theorizing of habitual body monitoring as an outcome of chronic self-objectification (e.g., Tiggemann and Slater, 2001), we conceptualize appearance monitoring as a process triggered by state self-objectification. We believe this conceptualization to be useful in highlighting that state self-objectification leads to attentional and cognitive load, resulting in impaired task performance.

^3^Evolutionary theorizing suggests that to attract potential mates, unattractive females can demonstrate that they offer qualities, such as cooperativeness, that males desire in long-term romantic relationships (Singh, 2004). This suggests that when women experience discrepancy from appearance standards, they may engage in stereotypically feminine behavior (e.g., present themselves as possessing more communal traits) in an attempt to ‘compensate’ for their lack of attractiveness. However, Saguy et al. (2010) found that objectified female participants did not present themselves as possessing more stereotypically feminine qualities. Therefore, our model does not include a path from experience of discrepancy from appearance standards to stereotype-consistent behavior.

## Appendix

**Table A1:** The existing experimental research on state self-objectification: a brief roadmap.

**Authors and publication year**	**Participants**	**Experimental design**	**Manipulation of state self-objectification**	**Outcome variables**
Fredrickson et al., 1998; Study 1	***N*** = 72 female undergraduates at Duke University ***M***_age_: not reported **Race/Ethnicity**: 70% Caucasian, 6% African American, 10% Asian, 7% Hispanic, 7% other ***M***_BMI_ = 22.57 **Sexual Orientation**: not reported **Family Status**: not reported	A two-cell experimental design (an objectifying vs. a non-objectifying condition); TSO measured as a potential moderator	Trying on a swimsuit vs. a sweater	**Affective responses:** body shame **Behaviors and motivations:** restrained eating
Fredrickson et al., 1998; Study 2	***N*** = 42 female and 40 male undergraduates at the University of Michigan ***M***_age_: not reported **Race/Ethnicity**: 83% Caucasian, 6% African American, 5% Asian, 2% Hispanic, 4% other ***M***_BMI_ = 23.43 for women, 24.84 for men **Sexual Orientation**: not reported **Family Status**: not reported	A 2 (condition: objectifying vs. non-objectifying) X 2 (gender: women vs. men) X 2 (high vs. low TSO)	Trying on a swimsuit vs. a sweater	**Manipulation check:** TST **Affective responses:** body shame, subjective report on emotions **Cognitive performance**: a math test
Gapinski et al., 2003	***N** =* 82 female undergraduates at Yale University ***M***_age_ = 18.69 **Race/Ethnicity**: 61% Caucasian, 14% Asian, 10% African American, 6% Hispanic, 9% other ***M***_BMI_ = 23.21 **Sexual Orientation**: not reported **Family Status**: not reported	A 2 (condition: objectifying vs. non-objectifying) X 2 (exposure to “fat talk” vs. control talk, which was also intended to manipulate objectification); TSO measured as a potential moderator	Trying on a swimsuit vs. a sweater; exposure to “fat talk” (a confederate saying “This [swimsuit/sweater] looks so horrible on me”) vs. “neutral talk”	**Manipulation check:** TST **Affective responses:** anxiety, negative emotions, self-efficacy **Behaviors and motivations:** intrinsic motivation to do well in the test **Cognitive performance:** a Gestalt Completion Test, a Non-sense Syllogisms Test, a Surface Development Test, a math test
Harrison and Fredrickson, 2003; Study 2	***N*** = 230 high-school and middle school female students in the Midwest (US) ***Range**_*age*_*: 10 to 17 ***M***_age_ = 13.00 **Race/Ethnicity**: 46.8-48.4% African American, 31.2-31.4% Anglo American, 3.3-6.5% Latina, 3.3% Asian American, 2% native American, 10.4-11.1% other. ***M***_BMI_*:* not reported **Sexual Orientation**: not reported **Family Status**: not reported	A 2 (race: White vs. non-White) X 3 (exposure to lean female athletes, non-lean female athletes, and male athletes) TSO was measured as a covariate ER: What is the meaning of the asterisk?	Watching sports media depicting lean female athletes (representing the White female idealized body), non-lean female athletes (representing the non-White idealized female body), or male athletes (a control condition)	TST (used as the primary DV)
Calogero, 2004	***N*** = 105 female undergraduates at a small Southeastern university (US) ***M***_age_ is not reported **Race/Ethnicity**: 83% Caucasian, 5% African American, 4% Asian American, 3% Hispanic, 5% other ***M***_BMI_*:* 21.71 **Sexual Orientation**: not reported **Family Status**: not reported	A three-cell experimental design (anticipating a male gaze, a female gaze or no gaze) TSO was measured as a covariate	Anticipating an interaction with a man, a woman, or no interaction	**Affective responses:** body shame, social physique anxiety **Behaviors and motivations:** dietary intentions
Hebl et al., 2004	***N*** = 224 female and 176 male students at two southern universities (US) ***M***_age_ = 21.89 **Race/Ethnicity**: 32.5% Caucasian, 23.25% African American, 22.5% Asian American, 22% Hispanic ***M***_BMI_*:* not reported **Sexual Orientation**: not reported **Family Status**: not reported	A 2 (condition: objectifying vs. non-objectifying) X 2 (gender: female vs. male) X 4 (race/ethnicity: African American, Caucasian, Asian American or Hispanic) TSO was measured as a covariate	Trying on a swimsuit (one-piece Speedo briefs for men) vs. a sweater	**Manipulation check:** TST **Affective responses:** body shame, state self-esteem **Behaviors and motivations:** restrained eating **Cognitive performance**: a math test
Roberts and Gettman, 2004	***N*** = 90 female and 70 male students at a Colorado public university ***M***_age_ = 19 **Race/Ethnicity**: 82% Caucasian, 15% Hispanic, 3% African American ***M***_BMI_*:* not reported **Sexual Orientation**: not reported **Family Status**: not reported	A 3 (self-objectifying prime, body competence prime and control) X 2 (gender: female vs. male)	Answering scrambled sentences test containing objectifying words (e.g., “sexy”), body competence words (e.g., “strong”) or neutral words (e.g., “silly”)	**Manipulation check:** TST **Affective responses:** shame, disgust, appeal of the physical aspects of sex, appearance anxiety
Fea and Brannon, 2006	***N*** = 185 female undergraduates at a large Midwestern university (US) ***M***_age_ = 18.80 **Race/Ethnicity**: 89% Caucasian, 5% Hispanic, 3% African American, 2% Asian American, 1% other ***M***_BMI_*:* not reported **Sexual Orientation**: not reported **Family Status**: not reported	A three-cell experimental design (type of compliment: neutral, character-related or appearance-related)	Receiving a character-related compliment (“You sound like a nice person”), appearance-related compliment (“You are a nice-looking person”), or no compliment from a 27-year old female experimenter	TST (used as a DV) **Affective responses:** negative mood, body shame, appearance anxiety
Quinn et al., 2006a	***N*** = 88 female and 62 male U.S. students ***M***_age_ = 18 **Race/Ethnicity**: not collected; participants were recruited from a pool with 85% Caucasian, 15% other ***M***_BMI_*:* women = 22.75, men = 25.04 **Sexual Orientation**: not reported **Family Status**: not reported	A 2 (condition: objectifying vs. non-objectifying) × 2 (gender: female vs. male)	Trying on a swimsuit vs. a sweater	**Manipulation check:** TST **Affect:** shame, body related thoughts
Quinn et al., 2006b	***N*** = 83 female participants in the US ***M***_age_ = 21.3 **Race/Ethnicity**: 26.5% Asian American, 25.3% Ethnicity: Caucasian, 25.3% Latin American, 22.8% African American ***M***_BMI_*:* not reported **Sexual Orientation**: not reported **Family Status**: not reported	A two-cell experimental design (condition: objectifying vs. non-objectifying)	Trying on a swimsuit vs. a sweater	**Manipulation check:** TST **Affective responses:** body shame **Cognitive performance**: a modified Stroop test
Choma et al., 2007; Study 2	***N*** = 366 female undergraduates at a university in Ontario ***M***_age_ = 18.65 **Race/Ethnicity:** 93.2% White Canadians, 6.8% other ***M***_BMI_* =* 22.64 **Sexual Orientation**: not reported **Family Status**: not reported	A two-cell experimental design (condition: objectifying [intervention movie] vs. non-objectifying) TSO was measured as a covariate	Watching a media literacy intervention movie (*Slim Hopes*), which criticizes the thinness ideal (“slim hopes”) vs. a neutral movie	**Manipulation check:** TST **Affective responses:** self-esteem (i.e., performance esteem, body esteem and social esteem), positive and negative affect
Johnson et al., 2007	***N*** = 90 male undergraduates at York University in Toronto ***M***_age_ = 21.49 **Race/Ethnicity:** not reported ***M***_BMI_ = 22.43 **Sexual Orientation**: not reported **Family Status**: not reported	A three-cell experimental design (condition: objectification of men, objectification of women, no objectification)	Watching neutral ads, ads objectifying men, or ads objectifying women	**Affective responses:** state self-esteem, drive for muscularity, psychological well-being (i.e., depression, general anxiety and hostility)
Martins et al., 2007; Study 2	***N*** = 57 Gay and 68 heterosexual male volunteers ***M***_age_: not reported, ***Range**_*age*_*: 17 to 40 **Race/Ethnicity:** not reported ***M***_BMI_*:* not reported **Sexual Orientation**: not reported **Family Status**: not reported	A 2 (condition: objectifying vs. non-objectifying) × 2 (sexual orientation: gay vs. straight)	Trying on Speedo briefs vs. a sweater	**Manipulation checks:** TST, a word-stem completion task (WST) assessing the activation of appearance-related schemas **Affective responses:** state body shame, state body dissatisfaction **Behaviors and motivations:** restrained eating **Other**: state body surveillance
Harper and Tiggemann, 2008	***N*** = 90 female undergraduates at Flinders University in Australia ***M***_age_ = 20.48 **Race/Ethnicity:** not reported ***M***_BMI_*:* 21.81 **Sexual Orientation**: not reported **Family Status**: not reported	A three-cell experimental design (condition: objectification of women vs. objectification of women and men vs. no-objectification). TSO was measured as a covariate	Exposure to advertisement featuring a thin woman (objectification of women condition), a thin woman with at least one attractive man (objectification of women and men condition), or no people (control condition)	**Manipulation check:** TST **Affective responses:** appearance anxiety, negative mood, body dissatisfaction
Tiggemann and Boundy, 2008	***N*** = 96 female undergraduates at Flinders University in Australia ***M***_age_ = 19.71 **Race/Ethnicity:** not reported ***M***_BMI_*:* not reported **Sexual Orientation**: not reported **Family Status**: not reported	A 2 (condition: objectifying vs. non-objectifying) × 2 (appearance comment: compliment vs. none); TSO measured as a potential moderator	Exposure to a subtle objectifying environment (i.e., scales and full-length mirrors) vs. a standard environment Receiving (vs. not receiving) an appearance compliment from a female experimenter (“I like your top”)	**Manipulation Check:** TST **Affective responses:** body shame, negative mood **Cognitive performance:** logical reasoning task, spatial orientation task
Aubrey et al., 2009	***N*** = 154 female undergraduates at a large Midwestern university (US) ***M***_age_ = 19.72 **Race/Ethnicity**: 84.4 %, Caucasian, 9.7% African American, 3.2% Asian American, 2.6% other ***M***_BMI_*:* not reported **Sexual Orientation**: not reported **Family Status**: not reported	A three-cell experimental design (condition: objectification through idealized bodies vs. objectification through segmented bodies vs. no objectification); Trait body-surveillance was measured as a potential moderator	Exposure to images of female models with high skin exposure (the objectification through idealized bodies condition), images of women segmented into body parts (the objectification through segmented body parts condition), or images unrelated to appearance of places and things (control condition)	TST (including each statement's valence, used as a DV), body—surveillance
Daniels, 2009	***N*** = 350 middle-school girls in California, ***M***_age_ = 14.96, ***Range***_age_ = 13-18, and 225 female undergraduates at a medium-sized California university, ***M***_age_ = 19, ***Range***_age_ = 18-22. **Race/Ethnicity**: 38 % Caucasian, 34% Latina, 20% mixed ethnicities, 5% Asian American, 1% Native American, 1% Black, 2% not reported. ***M***_BMI_*:* not reported **Sexual Orientation**: not reported **Family Status**: not reported	A 2 (condition: high vs. low sexualization) X 2 (targets of sexualization: athletes vs. models) experimental design	Exposure to media images featuring sexualized athletes or models (high objectification conditions), or non-sexualized (i.e., performance) athletes and models (low objectification conditions)	TST (used as the primary DV)
Gay and Castano, 2010; Study 1	***N*** = 25 U.S. female students (graduates and undergraduates) ***M***_age_: not reported **Range**_age_ = 18 to 35 **Race/Ethnicity:** not reported ***M***_BMI_*:* not reported **Sexual Orientation**: not reported **Family Status**: not reported	A two-cell experimental design (condition: high vs. low objectification); TSO measured as a potential moderator	Being videotaped from the neck down by either a man or a woman (representing high vs. low objectification conditions)	**Behaviors and motivations:** Appeal of physical aspects of sex **Cognitive performance:** Performance and response time in the LNS (letter number sequence test), a math test
Gay and Castano, 2010; Study 2	***N*** = 51 U.S. female students (graduates and undergraduates) ***M***_age_: not reported **Race/Ethnicity:** not reported ***M***_BMI_*:* not reported **Sexual Orientation**: not reported **Family Status**: not reported	A two-cell experimental design (condition: high vs. low objectification); TSO measured as a potential moderator	Being videotaped from the neck down by either a man or a woman	**Affective responses:** anxiety (self-reported), implicit and explicit self-esteem **Physiological responses:** skin conductance levels (SCL; used as an implicit measure of anxiety) **Cognitive performance:** Performance and response time in the LNS.
Saguy et al., 2010	***N*** = 114 female and 93 male undergraduates ***M***_age_ = 18.73 **Ethnicity**: not reported ***M***_BMI_*:* not reported **Sexual Orientation**: not reported **Family Status**: not reported	A 2 (gender: male vs. female) × 2 (partner's gender: male vs. female) × 3 (communication condition: body, face, audio)	Participants introduced themselves to a partner (another participant), knowing that s/he will listen to an audiotape of their introduction (in the control condition), watch a videotape of their introduction filmed from their neck up (in the low objectification condition), or from their neck down (in the high objectification condition)	**Manipulation check:** two items examining the extent to which participants felt “more like a body than as a real person” and as if their body and identity were separate things **Affective responses:** preference of experimental condition **Behaviors and motivations:** amount of talking when introducing themselves to an ostensible partner, self-presentation in terms of traditionally feminine/masculine traits
(Calogero and Pina, 2011); Study 2	***N*** = 85 female undergraduates at a southeastern British university ***M***_age_ = 21.89 **Race/Ethnicity**: 80.4% White, 4.8% Black African, 6% Asian, 8.8% Other/Mixed Race ***M***_BMI_ = 22.01 **Sexual Orientation**: 98.9% identified as heterosexual **Family Status**: not reported	A three-cell experimental design (body objectification, body empowerment, or body neutral)	Answering scrambled sentences test containing objectifying words (e.g., “sexy”), body competence words (e.g., “strong”), or neutral words (e.g., “silly”)	**Manipulation check:** TST **Affective responses:** body shame, body guilt **Behaviors and motivations:** eating restrains **Other:** body surveillance
Gervais et al., 2011	***N*** = 67 female and 83 male undergraduates at a large Midwestern university (US) ***M***_age_ = 19.16 **Race/Ethnicity**: 89% European American, 2.7% African American .67% Latino, 3.3% Asian American, 1.3% Multiracial, 2.7% other ***M***_BMI_: not reported **Sexual orientation**: 98% heterosexual, 1.3% bisexual, one lesbian **Family Status**: 48.7% single, 18.7% casual dating relations, 32.7% committed relationship	A 2 (gender: male vs. female) × 2 (condition: high vs. low objectification)	In the objectifying condition participants experienced an objectifying gaze from an interviewer (a trained confederate) of the opposite sex and received feedback that implicitly referred to their looks; in the non-objectifying condition the interviewer looked in their eyes during the interview and the feedback did not refer to their looks	**Manipulation check:** perception of partner's behavior **Affective responses:** body shame, body dissatisfaction **Behaviors and motivations:** motivation for future interaction with the partner **Cognitive performance**: a math test **Other**: body surveillance
Goldenberg et al., 2011; Study 1	***N*** = 122 female college students ***M***_age_ = 20.11 **Race/Ethnicity:** Caucasian ***M***_BMI_*:* not reported **Sexual Orientation**: not reported **Family Status**: not reported	A 3 (condition: objectifying, competence saliency, and neutral) X 2 (exposure vs. non-exposure to death prime); TSO measured as a potential moderator	Exposure to a cover of a magazine depicting a female model wearing a bikini, a full dressed female soccer player in motion, or a dining table	**Affective responses**: mood, level of liking of an image of an objectified woman
Goldenberg et al., 2011; Study 2	***N*** = 92 female college students ***M***_age_ = 21.85 **Race/Ethnicity**: 43% Caucasian, 21% Hispanic, 19% Black, 10% Asian, 7% other ***M***_BMI_*:* not reported **Sexual Orientation**: not reported **Family Status**: not reported	A 2 (appearance compliment vs. no compliment) X 2 (exposure vs. non-exposure to death prime); TSO measured as a potential moderator	Receiving (vs. not receiving) an appearance compliment from a female experimenter (“That's a really cute outfit”)	**Affective responses**: state self-esteem and body-esteem
Hopper and Aubrey, 2011	***N*** = 301 pregnant female volunteers ***M***_age_ = 29.20 **Race/Ethnicity**: 92.2% Caucasian, 2.6% African American, 2.3% Hispanic, 1.3% Asian Pacific Islander, 0.3% Native American, 1.0% other ***M***_BMI_*:* not reported **Sexual Orientation**: not reported **Family Status**: not reported	A three-cell experimental design (condition: high, low and no objectification); participants' stage in pregnancy, history with pregnancy, and age measured as potential moderators Habitual body surveillance was measured as a covariate	Exposure to images of pregnant celebrities that were highly sexualized (full body images), not sexualized (face only images), or images of baby products with no people featured	TST (used as the primary DV)
Thøgersen-Ntoumani et al., 2011	***N*** = 93 UK female undergraduates ***M***_age_ = 19.40 **Race/Ethnicity:** 86% White British origin, 5.37% Asian or Asian/British, 1.07% White European, 1.07% White other, 1.07% Black/Black British, 1.07 % Chinese, 1.07% mixed, 1.07% other. ***M***_BMI_ = 22.17 **Sexual orientation**: 98.40% heterosexual,1.60% bisexual **Family Status**: not reported	A two-cell experimental design (condition: high vs. low objectification); trait self-objectification, appearance evaluation and self-esteem measured as potential moderators	Trying on tight shorts and a sports bra-style top vs. baggy tracksuit trousers and a matching long-sleeved baggy top	**Manipulation check**: TST **Affective responses:** anxiety, depression, anger, happiness, confidence, feeling of fatness, physical attractiveness, body satisfaction
Green et al., 2012	***N*** = 28 Female undergraduates at a small Midwestern liberal arts college (US) ***M***_age_ = 19.30 **Race/Ethnicity**: 74.2% Caucasian, 6.5% African American, 6.5% Asian American, 3.2% Native American, 6.5% Biracial American,3.2% Other ***M***_BMI_*:* not reported **Sexual Orientation**: not reported **Family Status**: not reported	A within-participants experimental design (high vs. low objectification); TSO was measured as a potential moderator	Trying on a swimsuit vs. a tracksuit in a randomized order	**Physiological response:** mean heart rate (HR) at 6 s and 5 min after trying on the swimsuit and the tracksuit (measures related to women's clothing-related distress measures are not mentioned here because they were not compared across experimental conditions)
Prichard and Tiggemann, 2012	***N*** = 184 Australian female psychology undergraduates at Flinders University ***M***_age_ = 18.90 **Race/Ethnicity**: Caucasian ***M***_BMI_*:* not reported **Sexual Orientation**: not reported **Family Status**: not reported	A 2 (physical exercise vs. no exercise) × 2 (condition: high vs. low objectification); habitual exercising was measured as a potential moderator	Watching appearance-focused vs. neutral music videos while exercising vs. resting	Manipulation check: TSO (a modified version; examined before vs. after the manipulations) **Affective responses:** mood (before vs. after), body satisfaction (before vs. after)
Tiggemann and Andrew, 2012	***N*** = 102 Australian female undergraduates ***M***_age_ = 20.20 **Race/Ethnicity**: More than 90% were Caucasian ***M***_BMI_ = 21.92 **Sexual Orientation**: not reported **Family Status**: not reported	A within-participants experimental design of 2 (condition: high vs. low objectification) × 2 (setting: public vs. private); BMI and TSO were measured as potential moderators	Imagining oneself wearing either a swimsuit or a sweater, either in public or in a dressing room	state self-objectification (measured using the body-surveillance subscale of the Objectified Body Consciousness Scale; McKinley and Hyde, 1996) **Affective responses:** negative mood, body shame, body dissatisfaction
Calogero, 2013; Study 2	***N** =* 78 female undergraduates ***M***_age_ = 21.15 **Race/Ethnicity**: 91% Caucasian,9% African American **Sexual orientation**: 90% heterosexual, 8% bisexual, 2% lesbian **Family Status**: not reported	A two-cell experimental design (condition: objectifying vs. non-objectifying)	Writing about a time when one felt sexually objectified by another person vs. about what one would do with a $50 Target gift card	**Manipulation check: TST Behaviors and motivations:** gender-specific system justification, intention to engage in collective action to promote gender equality
Michaels et al., 2013	***N** =* 140 U.S. male undergraduates at a large Southeastern university (US) ***M***_age_ = 19.41 **Race/Ethnicity**: 58% White/European American, 14% Hispanic/Latino, 14% Asian American/Pacific Islander, 9% African American/ Black, 3% Biracial/Multiracial, 1% American Indian/Native American, 1% Arabic American/Middle Eastern, 1% Other ***M***_BMI_: not reported **Sexual orientation**: 64% exclusively heterosexual, 23% exclusively homosexual, 9% mostly homosexual, 4% mostly heterosexual,1% bisexual **Family Status**: not reported	A two-cell experimental design (condition: objectifying vs. non-objectifying); sexual orientation was measured as a potential moderator	Exposure to muscularity-idealizing media images (muscular shirtless men) vs. neutral images (animals, landscapes, and non-human objects)	**Affective responses:** body image (i.e., drive for muscularity, body dissatisfaction, body shame, and social physique anxiety) **Other:** body surveillance
Rollero, 2013	***N** =* 80 female and 86 male undergraduates ***M***_age_ = 24.5 **Race/Ethnicity**: Caucasian ***M***_BMI_ = 21.91 **Sexual Orientation**: not reported **Family Status**: not reported	A 2 (gender: men, women) x 3 (condition: objectification of men, objectification of women, no objectification)	Exposure to advertisements depicting objectified male models, objectified female models, or non-human objects	**Affective responses:** positive and negative mood (indicating subjective well-being), state self-esteem (including attractiveness, social, and performance subscales). **Behaviors and motivations**: ambivalent sexism toward women (ASI) and men (AMI)
Calogero et al., 2014	***N** =* 116 UK female college students ***M***_age_ * =* 18.78 **Race/Ethnicity**: 68% White, 18% Black, 14% Asian ***M***_BMI_: not reported **Sexual orientation**: 100% heterosexual **Family Status**: not reported	A three-cell experimental design (condition: sexually objectifying, non-objectifying physicality, or neutral content) Appearance control beliefs were measured as a covariate	Answering scrambled sentences test containing either sexually objectifying words (e.g., “sexiness”), words related to non-objectifying physicality (e.g., “health”), or neutral words (e.g., “car”)	**Manipulation check**: TST **Affective responses:** body shame **Behaviors and motivations:** intention to have a cosmetic surgery in the future (intrapersonal and social motives)
Fox et al., 2014; Study 1	***N** =* 87 female students at a large Midwestern university (US) ***M***_age_ * =* 20.04 **Race/Ethnicity**: 79.3% Caucasian/European American/White, 8% Black/African/African American, 6.9% Asian/Asian American, 2.2% Latina/Hispanic, 2.2% multiracial, 1.1% Middle Eastern ***M***_BMI_: not reported **Sexual orientation**: not reported **Family Status**: not reported	A 2 (watching vs. controlling an avatar) X 2 (avatar type: sexualized vs. non-sexualized) TSO was measured as a covariate	Controlling or watching sexualized (e.g., wearing exposed clothes) or non-sexualized female avatars using the Second *Life* virtual world	TST (used as the primary DV)
Fox et al., 2014; Study 2	***N** =* 81 female undergraduates at a large Midwestern university (US) ***M***_age_ * =* 19.91 **Race/Ethnicity**: 67.9% Caucasian/European American/White, 9.87% Black/African/African American, 8.64% Asian/Asian American, 7.4% multiracial, 6.17% other ***M***_BMI_: not reported **Sexual orientation**: not reported **Family Status**: not reported	A 2 (avatar type: sexualized vs. non-sexualized) X 2 (similar vs. dissimilar to the participant)	Controlling sexualized vs. non-sexualized female avatars	TST (used as a DV) **Behaviors and motivations:** endorsement of rape myths
Green et al., 2014	***N** =* 57 female and 53 male undergraduates at a small Midwestern college and volunteers from five surrounding communities (US) ***M***_age_ = 21.62 **Race/Ethnicity**:74.5% Caucasian, 6.4% African American, 3.6% Asian American, 5.5% Latinos/as, 0.9% Native American, 4.5% Biracial/Multiracial American, 3.6% Other, 0.9% nonrespondents ***M***_BMI_: not reported **Sexual orientation**: 91.8% heterosexual, 1.8% homosexual,6.4% bisexual **Family Status**: not reported	A 2 (gender: men vs. women) x 4 within-participants conditions (high vs. low objectification and two types of control) Baseline heart rate (HR) was measured prior to the assignment to experimental conditions	Following a measure of their baseline HR, participants tried on swimsuits while looking in the mirror (high-objectification), tracksuits while looking in the mirror (low-objectification), and perfume without looking in the mirror (no-objectification), and observed nature images (no-objectification). The order of these four conditions was randomized	**Affective responses:** positive and negative mood, body-related guilt **Physiological response:** HR at 6-s and 5-min intervals from the onset of each experimental manipulation
Kozak et al., 2014	***N** =* 80 female undergraduates in the Rocky Mountain West region (US) ***M***_age_*:* not reported, **Range**_age_ = 18–22 **Race/Ethnicity**: 79% European American/Caucasian, 10% Latina/Hispanic, 6% Asian American, 3% African American, 2% Native American/Pacific Islander ***M***_BMI_: not reported **Sexual orientation**: not reported **Family Status**: not reported	A 2 (condition: objectifying vs. non-objectifying) x 2 (posture: upright vs. slouched) x 2(Power: Type of chair: throne vs. child's) TSO was measured as a covariate	Trying on a tank top vs. a loose sweatshirt	**Manipulation check:** TST **Affective responses:** mood, satisfaction with one's task performance (see below) **Physiological response:** skin temperature (indicating stress level) **Cognitive performance**: Raven's Progressive Matrices task, a math test
Aubrey and Gerding, 2015	***N** =* 94 female undergraduates at a large Midwestern university (US) ***M***_age_ * =* 20.05 **Race/Ethnicity**: 56.8% White, 5.7% African American, 3.4% Hispanic/Latino, 2.3% Asian American, 31.8% other. Six participants did not report their race ***M***_BMI_: not reported **Sexual orientation**: not reported **Family Status**: not reported	A two-cell experimental design (condition: objectifying vs. non-objectifying)	Exposure to videos of female musicians that are either low or high in sexual objectification (sexual objectification operationalized as degree of body exposure, close-up shots of sexual body parts, and suggestive dance, moves, or gestures in the presence of a male audience)	**Manipulation check:** TST **Cognitive Performance**: cognitive processing (encoding, recognition and recall of factual information) of subsequent television commercials
Ford et al., 2015; Study 1	***N** =* 49 female and 50 male undergraduates at two Southeastern universities (US) ***M***_age_ * =* 19.90 **Race/Ethnicity**: 85.85% Whites, 3.03% African-Americans, 6.06% Asians, 4.04% Hispanics, 1.17% did not indicate their race ***M***_BMI_: not reported **Sexual orientation**: not reported **Family Status**: not reported	A 2 (gender: men vs. women) x 2 (condition: sexist and objectifying vs. non-sexist and non-objectifying)	Rating the degree of funniness of four comedy clips, which were either sexist and objectifying (portraying women as sex objects whose value is derived from physical appearance and depicting women as inferior to men) or neutral	State self-objectification (using a modified version of the Self-Objectification Questionnaire, SOQ)
Ford et al., 2015; Study 2	***N** =* 57 female volunteers from the U.S ***M***_age_ = 35.73 **Race/Ethnicity:** 75.43% Whites, 14.03% African Americans, 3.50% Asians, 3.50% multi-racial people, 1.75% other (one woman did not indicate her race) ***M***_BMI_: not reported **Sexual orientation**: not reported **Family Status**: not reported	A two-cell experimental design (condition: sexist, objectifying vs. non-sexist, non-objectifying clips)	Watching sexist and objectifying vs. neutral comedy clips in an imagined group setting (a role playing task)	TST (used as a DV) **Other:** body surveillance
Krawczyk and Thompson, 2015	***N** =* 310 female and 127 male students at a large Southeastern university (US) ***M***_age_ = 19.98 **Race/Ethnicity**: 48.3% White, 16.9% Hispanic/Latino, 16.5% African American, 10.5% Asian American, 7.3% bi-racial or other, 0.2% Native American, and 0.2% Pacific Islanders ***M***_BMI_: not reported **Sexual orientation**: 94.7% heterosexual, 3.4% bisexual, 1.8% gay or lesbian **Family Status**: 56.1% single, 39.6% in a relationship, 2.7% engaged, 0.9% married, and 0.7% other	A 2 (gender: men vs. women) x 2 (condition: objectifying vs. non-objectifying) experimental design; internalization of cultural appearance ideals was measured as a potential moderator	Watching advertisements that sexually objectify women and portray appearance ideals vs. neutral advertisements	**Affective responses:** body- dissatisfaction **Other:** ratings of women's attractiveness and competence
Register et al., 2015	***N** =* 100 female and 54 males (two did not report their gender) students at a community college in California and a public university in Missouri ***M***_age_ = 22.01 **Race/Ethnicity**: 37% White, 32% African American, 15% Asian, 5%, 11% other ***M***_BMI_ = 26.31 **Sexual orientation**: not reported **Family Status**: not reported	A 2 (gender: men vs. women) x 2 (condition: objectifying vs. non-objectifying)	Describing oneself from someone else's perspective (“Explain how this other person sees you and compares your body to the “ideal” body for your gender”) vs. writing about one's activities over the last 24 h	**Manipulation check:** state self-objectification (using a modified version of the SOQ) **Affective responses:** depressive symptoms **Behaviors and motivations:** self-reported eating pathology (measured using the Drive for Thinness subscale of the Eating Disorder Inventory)
Hopper and Aubrey, 2016	***N** =* 127 female students at a public Midwestern university (US) who were never pregnant ***M***_age_ = 20, **Range**_age_ = 18-24 **Race/Ethnicity**: 78% White, 10.2% African American, 3.9% Asian, 5%, Hispanic, 3.1% did not report their race ***M***_BMI_ = 23.05 **Sexual orientation**: not reported **Family Status**: not reported	A three-cell experimental design (condition: image of a post-pregnancy celebrity's full body/ image of a post-pregnancy celebrity's face/ image of home décor)	Participants in the objectification conditions were exposed to covers of magazines depicting images of either the full body or the face of celebrities after giving birth, with the title “celebrity after baby. ” The control condition depicted a house décor image	**Manipulation check**: TST and the body surveillance subscale of the OBCS
Guizzo and Cadinu, 2016	***N** =* 107 Italian women ***M***_age_ = 21.23 **Race/Ethnicity**: Caucasian ***M***_BMI_ = not reported **Sexual orientation**: 90% heterosexuals, 3% lesbians, 5% bisexuals, 2% did not indicate their sexual orientation **Family Status**: not reported	A two-cell experimental design (condition: female gaze vs. male gaze); internalization of cultural appearance ideals was measured as a potential moderator	Being photographed from the neck down by either a man or a woman (representing high vs. low objectification conditions)	**Cognitive performance:** sustained attention to response task **Other**: body- surveillance, self-perception of competence, morality and warmth, experience of flow, task intrusive thoughts
Fisher et al., 2017	***N** =* 127 female students at a small liberal art university (US) ***M***_age_ = 19.02, **Range**_age_ = 18-22 **Race/Ethnicity**: White, 58.7%, Hispanic/Latina 18.5%, African American, 15.2%, Asian, 3.3%, Native America 2.2% and Other 2.2%. ***M***_BMI_ = not reported **Sexual orientation**: not reported **Family Status**: not reported	A two-cell experimental design (condition: being exposed to catcalling scenario videos vs. a control condition). TSO, trait body image, previous objectification experiences and pretest dependent variable scores for objectification and body image were measured as covariates.	Watching a catcalling video which included four women being catcalled by a man while they walked down the street. vs. a control video where the same women walked on the street without the catcalling	TST (used as a DV) **Affective responses:** body image
Linder and Daniels, 2017	***N** =* 227 female and 193 male students at a liberal art college in the Northwest and a large Western university (US) ***Women M***_age_ = 19.73, ***Men M***_age_ = 19.56, ***Range***_age_ = 18-25 ***M***_BMI_ = not reported **Race/Ethnicity**: 72.3% White, 12.1% Hispanic, 5.4% Asian, 3.1% Black, 5.2% other **Sexual orientation**: All participants were heterosexual **Family Status**: not reported	A 2 (gender: men vs. women) x 2 (condition: objectifying vs. non-objectifying); Participants' peers preoccupation with body appearance and athlete engagement were measured as potential moderators	Exposure to sexualized vs. non-sexualized (i.e., performance) pictures of same-sex athletes (women viewed female athletes; men viewed male athletes)	TST (used as the primary DV, coded as physicality self-descriptors or self-objectifying statements)
Loughnan et al., 2017; Study 1	***N** =* 114 women recruited via Mturk ***M***_age_ = 36.96 ***M***_BMI_ = not reported **Race/Ethnicity**: 84% Caucasian, 9% African American, 5% Asian, 2% other **Sexual orientation:** not reported **Family Status**: not reported	A 2 (self- perception: baseline vs. objectified) x 2(observer's gender: male vs. female) x 2(observer's closeness: known vs. unknown) mixed factorial experimental design (with self-perception as a within-subjects factor)	Writing about a time when one felt objectified by another person. The other person was either male or female, and was either known or unknown to the participant	Other: self-perception of humanity (nature and uniqueness), warmth, competence and morality
Prichard et al., 2017	***N** =* 152 Australian women ***M***_age_ = 21.55, ***Range***_age_ = 17-30 ***M***_BMI_ = 23.81, ***Range***_BMI_ = 17.26-55.71 **Race/Ethnicity**: 92.1 % White, 2% Asian, 2% Indigenous Australian, 3.9% other **Sexual orientation**: not reported **Family Status**: not reported	A 2 (presentations of the body: functional vs. non-functional) × 2 (appearance focused text: with vs. without text) × 2 (time: pre-exposure, post-exposure) mixed design. TSO was examined as a moderator	Viewing inspirational fitness images (“ fitspiration”) which depicted the body in a functional vs. non-functional way (i.e., performing exercises vs. posing); exposure vs. no exposure to an appearance-focused text	State self-objectification (using the SOQ pre and post manipulation, used as a DV) **Affective responses:** state body satisfaction, negative mood
Jiang, 2018	***N** =* 120 Chinese female students at a large Southwestern university in China ***M***_age_ = 20.68, ***Range***_age_ = 18-25 ***Range***_BMI_ = 18.50-22.06 **Race/Ethnicity**: Chinese **Sexual orientation:** not reported **Family Status**: not reported	A 2 (revealing nature of the clothing: revealing vs. full) × 2 (tightness of the clothes: tight-fitting vs. loose) × 2 (setting: private vs. public) mixed design. TSO and BMI were measured as covariates	Trying on one of four suits: revealing-loose, revealing-tight, full-loose or full-tight; Setting—personal fitting room vs. public study lounge—manipulated within participants	The body surveillance subscale of the OBCS was measured twice, once in each of the two settings (used as the primary DV)
Kahalon et al., 2018a; Study 1	***N** =* 88 Israeli female students at a large university in Israel ***M***_age_ = 23.61 ***M***_BMI_ = 21.77 **Race/Ethnicity**: Israeli Jewish **Sexual orientation**: 92% heterosexual, 4% bisexual, 1% gay, 3% did not respond. **Family Status**: not reported	A three-cell experimental design (type of compliment: achievement-related, appearance-related or no compliment). TSO was measured as a moderator	Writing about a neutral (no compliment) situation or a situation in which a man with whom they were not in an intimate relationship complimented either their academic/professional achievements or their appearance	**Manipulation check:** The body surveillance subscale of the OBCS was measured in a pilot study **Cognitive performance:** a math test
Kahalon et al., 2018a; Study 2	***N** =* 73 female and 75 male students at a large university in Israel ***M***_age_ = 23.79 ***M***_BMI_ = 22.48 **Race/Ethnicity**: Israeli Jewish **Sexual orientation**: 94% heterosexual, 4% gay, 1% bisexual, 1% did not respond. **Family Status**: not reported	A 2 (gender: men vs. women) x 2 (condition: appearance compliment vs. no compliment). TSO was measured as a moderator	Receiving feedback on their curriculum vitae, ostensibly written by a vocational counselor, which either or not complimented their “presentable appearance”	**Affective responses**: mood **Cognitive performance**: a math test

*The papers are ordered by year of publication; within each year, papers are ordered alphabetically, according to the first author's last name. Included in the list are experimental papers that manipulated state self-objectification, searched through Google Scholar and the PsycNET database. We used the following abbreviations: TSO for trait self-objectification, SOQ for self-objectification questionnaire, TST for the Twenty Statements Test, UK for United Kingdom, US for the United States and Mturk for Amazon's Mechanical Turk. The table mentions only manipulation checks that examined state self-objectification but no other variables [e.g., priming death in Goldenberg et al's (2011) study]*.
